# The ESAT-6 Protein of *Mycobacterium tuberculosis* Interacts with Beta-2-Microglobulin (β2M) Affecting Antigen Presentation Function of Macrophage

**DOI:** 10.1371/journal.ppat.1004446

**Published:** 2014-10-30

**Authors:** Gopalkrishna Sreejit, Asma Ahmed, Nazia Parveen, Vishwanath Jha, Vijaya Lakshmi Valluri, Sudip Ghosh, Sangita Mukhopadhyay

**Affiliations:** 1 Laboratory of Molecular Cell Biology, Centre for DNA Fingerprinting and Diagnostics (CDFD), Nampally, Hyderabad, India; 2 Division of Immunology and Molecular Biology, LEPRA Society-Blue Peter Research Centre, Hyderabad, India; 3 Molecular Biology Unit, National Institute of Nutrition (ICMR), Jamai-Osmania Hyderabad, India; Portland VA Medical Center, Oregon Health and Science University, United States of America

## Abstract

ESAT-6, an abundantly secreted protein of *Mycobacterium tuberculosis* (*M. tuberculosis*) is an important virulence factor, inactivation of which leads to reduced virulence of *M. tuberculosis*. ESAT-6 alone, or in complex with its chaperone CFP-10 (ESAT-6:CFP-10), is known to modulate host immune responses; however, the detailed mechanisms are not well understood. The structure of ESAT-6 or ESAT-6:CFP-10 complex does not suggest presence of enzymatic or DNA-binding activities. Therefore, we hypothesized that the crucial role played by ESAT-6 in the virulence of mycobacteria could be due to its interaction with some host cellular factors. Using a yeast two-hybrid screening, we identified that ESAT-6 interacts with the host protein beta-2-microglobulin (β2M), which was further confirmed by other assays, like GST pull down, co-immunoprecipitation and surface plasmon resonance. The C-terminal six amino acid residues (90–95) of ESAT-6 were found to be essential for this interaction. ESAT-6, in complex with CFP-10, also interacts with β2M. We found that ESAT-6/ESAT-6:CFP-10 can enter into the endoplasmic reticulum where it sequesters β2M to inhibit cell surface expression of MHC-I-β2M complexes, resulting in downregulation of class I-mediated antigen presentation. Interestingly, the ESAT-6:β2M complex could be detected in pleural biopsies of individuals suffering from pleural tuberculosis. Our data highlight a novel mechanism by which *M. tuberculosis* may undermine the host adaptive immune responses to establish a successful infection. Identification of such novel interactions may help us in designing small molecule inhibitors as well as effective vaccine design against tuberculosis.

## Author Summary

*M. tuberculosis* is a dangerous and highly successful pathogen that has evolved several mechanisms to manipulate the host immune regulatory network. Proteins secreted by *M. tuberculosis* play important roles in virulence. One such protein is ESAT-6, which is secreted along with its chaperone CFP-10. Despite a host of studies highlighting modulation of immune responses by ESAT-6, there have not been many that identified host proteins interacting with ESAT-6. We have now found that the host protein β2M interacts very specifically with ESAT-6 at its C-terminal region. The soluble ESAT-6:CFP-10 complex was found to be trafficked into the endoplasmic reticulum, and treatment with recombinant ESAT-6:CFP-10 or the over-expression of ESAT-6 reduced cell surface expression of β2M and molecules which remain associated with it like HLA-I. Recombinant ESAT-6:CFP-10 was also found to reduce classical and cross presentation of peptide antigens by MHC-I molecules. In summary, our data indicate that interaction between ESAT-6 and β2M can reduce the levels of available free β2M that associate with HLA/MHC-I molecules. This could be an interesting mechanism by which *M. tuberculosis* inhibits classical and cross presentation of peptide antigens in order to prevent or delay the onset of anti-mycobacterial adaptive immune responses.

## Introduction

Tuberculosis (TB) has remained a major cause of death worldwide despite the availability of a vaccine and several chemotherapeutic agents [1]. Global emergence of multi-drug resistant strains of *Mycobacterium tuberculosis* besides synergy with HIV [1] warrants a better understanding of the host-*M. tuberculosis* interaction in order to develop effective strategies for containing the disease. Successful infection of macrophages by pathogenic mycobacteria involves modulation of several immune functions, which allows them to survive and persist inside the host [2]. Crucial host cell functions required for development of anti-mycobacterial immunity like phagosome-lysosome fusion, autophagy, antigen presentation, apoptosis and bactericidal innate immune responses are known to be inhibited by the pathogenic mycobacteria [2]. Particularly, the proteins secreted by virulent mycobacteria are found to play important roles in modulation of host immune responses [3]. In addition to the classical Sec and Tat secretion pathway, *M. tuberculosis* possesses the ESX secretion system, a Type VII secretion system (T7SS) which is found to be non-essential for growth *in vitro*, but is required for bacterial virulence [4]. Mycobacteria possess at least five ESX secretion systems, namely ESX-1 through ESX-5 [5]. The first and the most characterized member of the ESX secretion system family, ESX-1 is encoded by region of difference (RD) 1 which is absent in all vaccine strains of avirulent *M. bovis* BCG, but present in the virulent laboratory and clinical strains of *M. bovis* and *M. tuberculosis* [5]. The RD1 region is also absent in *M. microti* which seldom causes disease in immunocompetent individuals [6]. Interestingly, deletion of RD1 results in attenuation of *M. tuberculosis* and it is hence implicated to play an important role in mycobacterial pathogenicity [4], [7]. RD1 complemented avirulent *M. bovis* BCG and *M. microti* strains were shown to grow better in severe combined immunodeficient mice and persist longer in organs of immunocompetent mice corroborating the fact that deletion of RD1 resulted in attenuation of these strains [8]. Mutants defective for ESX-1 secretion system are found to exhibit a range of diverse phenotypes, including defects in immune modulation, tissue invasion, phagosomal trafficking and growth inside the macrophages [4], [9], [10].

In *M. tuberculosis*, the early secretory antigenic target (ESAT)-6 or Rv3875 and the culture filtrate protein (CFP)-10 or Rv3874 are abundantly produced and secreted in culture. They are also known to be the most immunogenic molecules found in the culture filtrate [11], [12]. These proteins lack the classical Sec YEG secretory signal sequences and their secretion was found to be dependent on ESX-1 [4], [8], [10]. ESAT-6 and CFP-10 together form a tight dimer and are dependent on each other for their stability and secretion [9], [13], [14]. Along with the ESX-1 secretion system, ESAT-6 and CFP-10 have been implicated in several virulence mechanisms of mycobacteria. They are capable of modulating both innate and adaptive immune responses and inactivation of ESAT-6 results in dramatically reduced virulence of *M. tuberculosis* [4], [8], [9]. Interestingly, specific mutations in ESAT-6 that did not affect functioning of the ESX-1 secretion system caused a loss of virulence of mycobacteria [13], [15]. This implies that ESAT-6 itself is crucial in mediating the virulence functions associated with the ESX-1 secretion system. ESAT-6 was found to induce apoptosis of macrophages by activating caspase expression [16]. ESAT-6, CFP-10 and the ESAT6:CFP-10 complex can inhibit LPS-induced NF-kappaB dependent gene expression by suppressing production of reactive oxygen species [17]. ESAT-6 alone or in complex with CFP-10 has also been shown to interact with host proteins like laminin on the basolateral surface of pneumocytes leading to lysis of these cells that aid in the dissemination of *M. tuberculosis* in the human lung [18]. Moreover, ESAT-6 has been shown to interact directly with toll like receptor (TLR) 2 resulting in reduced interleukin (IL)-12 p40 secretion in macrophages, probably favouring a T helper 2 phenotype that helps intracellular persistence and survival of *M. tuberculosis* [19]. However, the exact mechanism of virulence of ESAT-6 is not well understood.

It has been shown that at lower pH, characteristic of the acidic phagosomal environment, the secreted ESAT-6:CFP-10 complex can dissociate and once dissociated, ESAT-6 alone can destabilize and lyse the phagosomal membrane [20]. Therefore, it appears that the ESAT-6:CFP-10 complex provides the bacteria or its components the tools necessary to escape from the phagosomal compartments to interact with the host cellular proteins and modulate their functions [20]. Based on the solution structure, no DNA binding or enzymatic function could be ascribed to the ESAT-6:CFP-10 complex [14] as the surface of the complex was found to be devoid of any acidic or basic patches characteristic of DNA-binding proteins and many transcription factors. Similarly, absence of significant clefts on the surface of the structure, indicative of an enzyme active site, suggests a non-catalytic role for this complex. Therefore, we hypothesized that the crucial role played by ESAT-6 in the virulence of mycobacteria could be due to its interaction with some host cellular factors, the identification of which is likely to provide better understanding of the role played by ESAT-6 in modulation of host immune responses. Accordingly, the aim of the present study was to identify ESAT-6 interacting partners of host origin by employing a yeast two hybrid assay system. Here, we report that ESAT-6 can specifically interact with beta-2-microglobulin (β2M), a small ∼13 kDa protein found to be associated with major histocompatibility complex (MHC) class I family of proteins that are involved in antigen presentation and iron regulation. We found that ESAT-6 was able to enter the endoplasmic reticulum (ER), where it interacts with β2M and inhibits its association with MHC-I molecules leading to reduced surface expression of MHC-I-β2M complex and consequently inhibits loading of antigen-derived peptides to the MHC-I complex. Data presented in this study highlight the existence of a novel mechanism by which *M. tuberculosis* ESAT-6 protein exerts its virulence by undermining the host adaptive immune responses to establish a successful infection and these findings may aid in the development of novel therapeutics against the deadly disease.

## Results

### ESAT-6 interacts with human β2M

**Figure 1:**
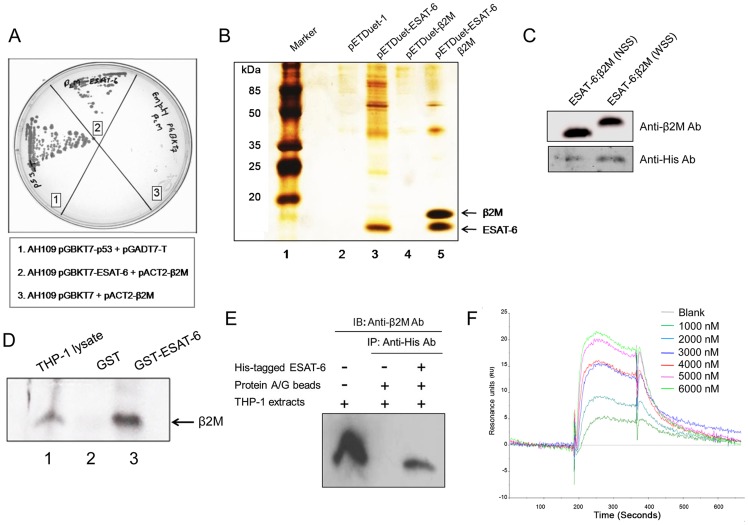
ESAT-6 protein interacts with β2M. (A) Interaction between ESAT-6 and β2M in a yeast two-hybrid system was studied by mating yeast strain AH1109 transformed with bait plasmid pGBKT7-ESAT-6 with yeast strain Y187 transformed with a human leukocyte cDNA prey library on a synthetic dropout plate (–Ade/–His/–Leu/–Trp). Prey cDNA was amplified by PCR using primers encompassing the cDNA insert in pACT2 and sequenced and identified to be β2M. The AH109 yeast strain transformed with pGBKT7-ESAT-6 and pACT2-β2M shows Ade and His interaction reporter activation on QDO plates. AH109 transformed with pGBKT7 and pACT2-β2M was used as a negative control while AH109 transformed with pGBKT7-p53 and pGADT7-T was used as a positive control for the yeast two-hybrid screening. (B) Untagged β2M was cloned along with His-tagged ESAT-6 in the pETDuet vector system and was transformed into *E. coli* BL21 cells. The transformed cultures were induced with IPTG and the over-expressed proteins were purified using TALON resin. Purified proteins were separated on a 16% Tris-Tricine SDS-PAGE and visualized by silver staining. Lane 1 is a molecular weight marker. (C) β2M with no N-terminal signal sequence (ESAT-6:β2M NSS) and the full length β2M with the N-terminal signal sequence (ESAT-6:β2M WSS) were cloned in pETDuet vector along with 6× His-tagged ESAT-6. Clones were over-expressed in *E. coli* BL21. Protein expression was induced by addition of IPTG and over-expressed proteins were purified using metal affinity TALON resin. Purified proteins were separated on 16% Tris-Tricine SDS-PAGE and transferred onto nitrocellulose membranes and probed with anti-β2M Ab to detect β2M and anti-His Ab to detect ESAT-6. The bands corresponding to β2M (upper panel) and ESAT-6 (lower panel) were visualized by chemiluminescence. (D) GST pre-cleared THP-1 macrophage extract was mixed and incubated with Glutathione-agarose bead bound-GST or GST-ESAT-6 and washed. The bound proteins were eluted and resolved on a 16% Tris-Tricine SDS-PAGE gel and immunoblotted for β2M protein using rabbit anti-human β2M Ab and HRP conjugated anti-rabbit Ab. Blots were visualized by chemiluminescence. Whole cell extracts from THP-1 cells was used as positive control for β2M expression. (E) In another experiment, purified recombinant 6× His-tagged ESAT-6 was incubated with THP-1 macrophage lysate and immunoprecipitated (IP) with anti-His Ab bound to protein A/G agarose beads. Control immunoprecipitation was carried out without the addition of His-tagged ESAT-6 protein (IP control). The eluted protein mixture is resolved on a 16% Tris-Tricine SDS-PAGE gel and immunoblotted (IB) for β2M protein using a rabbit anti-human β2M Ab and HRP conjugated anti-rabbit secondary Ab and the blots were visualized by chemiluminescence. Lane 1 is input control. (F) Direct interaction of β2M with ESAT-6 was monitored using a BIACORE 3000 Biosensor where β2M was immobilized on the sensor chip and recombinant ESAT-6 at different concentrations was injected in the running buffer. The changes in the refraction index at the surface due to interactions between immobilised β2M and fluid phase ESAT-6 were detected and recorded as RU (Resonance Units). Curves generated from the RU trace were evaluated using a curve-fitting algorithm. ESAT-6 was found to bind specifically to β2M and no binding was observed in a control cell which did not have any immobilized β2M. Results are representative of three different experiments.

We carried out yeast two-hybrid (Y2H) screening to identify the probable ESAT-6 interacting proteins from the host. ESAT-6 cloned in the bait vector pGBKT7 was used to screen a human leukocyte cDNA library cloned into the prey vector pACT2. The suitability of ESAT-6 for use as bait was confirmed by measuring its expression as GAL4-ESAT-6 fusion protein and also by assessing the toxicity of the fusion protein as well as auto-activation of interaction markers. Yeast strain AH109 expressing the Gal4-ESAT-6 fusion protein had normal growth kinetics and auto-activation of the reporter genes was not observed in these transformants. For Y2H screening, Mat-a strain AH109 harboring the bait vector pGBKT7-ESAT-6 was mated with Mat-α strain Y187 transformed with prey library plasmid and the mating mixture was plated on QDO plates (SD/–Ade/–His/–Leu/–Trp) for high stringency of selection. The prey plasmids, rescued from the colonies that appeared on selection plates, were sequenced using 3′ AD Sequencing Primer and were identified by querying these sequences against the NCBI GenBank database using the MegaBlast program. One of these cDNA sequences in the prey plasmid was found to have very high similarity with human beta-2-microglobulin (Figure S1). The interaction was found to be truly positive as the prey plasmid carrying the β2M cDNA sequence (pACT2-β2M) failed to grow in the presence of empty bait vector pGBKT7 alone (Figure 1A).

The physical interaction between ESAT-6 and β2M was confirmed by a co-purification assay. ESAT-6 was cloned into the first multiple cloning site (MCS) with an N-terminal His-tag and β2M cDNA from the prey plasmid was cloned into the second MCS without any tag in a dual expression vector pETDuet-1. When the over-expressed His-tagged ESAT-6 protein was purified from the transformed *Escherichia coli* using TALON affinity binding resin, β2M was found to be co-purified along with His-tagged ESAT-6, indicating a positive interaction between ESAT-6 and β2M (Figure 1B, Lane 5). β2M is known to possess a 20 amino acid endoplasmic reticulum (ER) localization sequence at its N-terminal which is removed from the mature protein during post-translational modifications. Since the prey plasmid cDNA encoding β2M contained the signal peptide sequence, any interaction with ESAT-6 through this region would be most likely physiologically irrelevant. Therefore, we examined whether ESAT-6 could actually interact with the mature β2M devoid of the signal peptide. The N-terminal signal sequence (20 amino acids) of β2M was therefore truncated and co-expressed along with His-tagged ESAT-6. It was found that the mature β2M could be co-purified along with His-tagged ESAT-6 (Figure 1C) indicating that the N-terminal signal peptide of β2M is not involved in the interaction with ESAT-6 protein.

We also examined whether ESAT-6 could interact with the β2M naturally present within mammalian cells. For this, GST-tagged ESAT-6 was used to pull down β2M from the whole cell extract prepared from PMA-differentiated THP-1 macrophages. It was found that the GST-ESAT-6 fusion protein was able to pull down β2M while GST alone failed to do so (Figure 1D) indicating that ESAT-6 can physically interact with mature β2M naturally expressed in macrophages. To further confirm this observation, a co-immunoprecipitation (Co-IP) assay was carried out where recombinant His-tagged ESAT-6 protein was incubated with THP-1 whole cell lysate and then anti-His antibody (Ab) coupled to protein A/G beads was added to the mixture to immunoprecipitate the interacting complex. The immunoprecipitated complex was separated by SDS-PAGE followed by immunoblotting using anti-human β2M Ab. A β2M specific band was detectable in the His-tagged ESAT-6-immunoprecipitated complex indicating that ESAT-6 interacts with β2M present in the THP-1 macrophages (Figure 1E). Physical interaction of ESAT-6 and β2M was further confirmed by using surface plasmon resonance (Figure 1F). The Kd value of the ESAT-6:β2M complex as determined from the surface plasmon resonance data was 1.03×10^−6^ M.

### β2M interacts with the C-terminal extreme of ESAT-6

**Figure 2:**
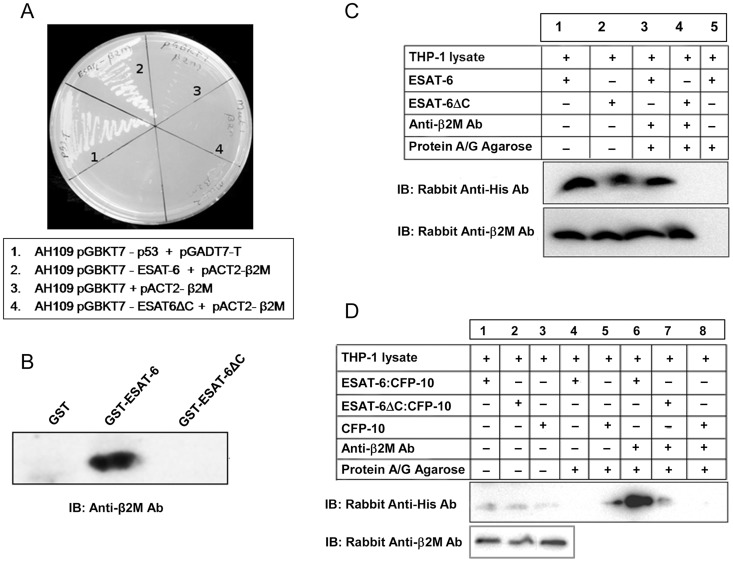
ESAT-6 with a deletion of six C-terminal amino acids (ESAT-6ΔC) fails to interact with β2M. (A) Yeast strain AH109 transformed with ESAT-6/ESAT-6ΔC bait along with β2M prey plasmid were streaked on interaction selection QDO plates (SD/–Ade/–His/–Leu/–Trp) along with the controls and monitored the growth resulting from positive interactions. (B) ESAT-6 or ESAT-6ΔC expressed as GST fusion protein was incubated with THP-1 lysate and precipitated using glutathione-agarose. Presence of β2M in the precipitated complexes was detected by Western blotting using anti-β2M Ab. (C) Recombinant His-tagged ESAT-6 or His-tagged ESAT-6ΔC protein was mixed and incubated with THP-1 cell extracts and β2M was immunoprecipitated using mouse anti-human β2M Ab and protein A/G beads. Presence of ESAT-6/ESAT-6ΔC and β2M in the immunoprecipitated complexes were detected by Western blotting using rabbit anti-His Ab and rabbit anti-human β2M Ab respectively. Lanes 1 and 2 are input controls. (D) THP-1 macrophage whole cell extract was mixed and incubated with recombinant ESAT-6:CFP-10 or mutant ESAT-6ΔC:CFP-10 or CFP-10 protein. β2M was immunoprecipitated using protein A/G bound mouse anti-human β2M Ab and the immune complexes were subjected to Western blotting using rabbit anti-His Ab to detect ESAT-6:CFP-10 or mutant ESAT-6ΔC:CFP-10 or CFP-10. The lanes 1–3 are input controls for these recombinant proteins (upper panel). In the lower panel, the lanes 1–3 indicate the levels of β2M in the input controls as determined by Western blotting using rabbit anti-human β2M Ab.

The extreme C-terminus of ESAT-6 is a floppy, structurally ill defined region, away from the helical core and is not known to be involved in the interaction with CFP-10 [14]. Mutant *M. tuberculosis* expressing a truncated ESAT-6 having residues deleted from the C-terminal end was found to be attenuated. However, these mutant strains had a functional ESX-1 system with normal secretion of the ESAT-6:CFP-10 complex [13], [15] indicating that the residues at the C-terminal end of ESAT-6 are not required for a structural role in the formation of a heterodimer with CFP-10 but crucial for the virulence functions of ESAT-6. Therefore, we speculated that the free C-terminus of ESAT-6 is probably involved in the interaction with β2M and such an interaction is possibly critical for *M. tuberculosis* virulence [21]. Once again using the yeast two hybrid assay, we observed that deletion of the last 6 amino acids (valine, threonine, glycine, methionine, phenylalanine and alanine) from the C-terminal end of ESAT-6 (Figure S2) was sufficient to prevent the interaction of ESAT-6 and β2M (Figure 2A). To further validate the yeast two hybrid data, we used a C-terminally truncated ESAT-6 (ESAT-6ΔC) to carry out GST pull down assay. The GST-tagged full length ESAT-6 protein (ESAT-6-GST) as well as the GST-tagged ESAT-6ΔC (ESAT-6ΔC-GST) protein were incubated with THP-1 extract and the pulled down fractions were probed for the presence of β2M using anti-β2M Ab. It was found that only the full length ESAT-6 protein was able to interact with β2M (Figure 2B). To further confirm our observations, we carried out a Co-IP assay where THP-1 lysates were incubated with full length His-tagged ESAT-6 or His-tagged ESAT-6ΔC and β2M-complexes were immunoprecipitated using anti-β2M Ab. The immunoprecipitated complexes were resolved on SDS-PAGE and probed with either anti-His Ab to detect ESAT-6/ESAT-6ΔC (Figure 2C, upper panel) or anti-β2M Ab to detect β2M (Figure 2C, lower panel). The data shown in Figure 2C clearly demonstrate that anti-β2M antibody can immunoprecipitate the full length ESAT-6 (Figure 2C, Lane 3), but not the mutant ESAT-6ΔC (Figure 2C, Lane 4), indicating that the C-terminal (90–95) residues of ESAT-6 are important for its interaction with β2M. Lane 5 of Figure 2C is negative control where Protein A/G beads were used to rule out any non-specific adsorption to the beads. Lanes 1 and 2 of Figure 2C are input controls showing the presence of β2M (lower panel) and ESAT-6 (Lane 1, upper panel) or ESAT-6ΔC (Lane 2, upper panel) in the reaction mixture containing THP-1 lysate and His-tagged ESAT-6 or ESAT-6ΔC.

ESAT-6 is predominantly secreted by *M. tuberculosis* as a tight 1∶1 heterodimeric complex with CFP-10 through the ESX-1 secretion system [22]. Earlier we found that the C-terminal end of ESAT-6 (amino acid residues 90–95) is involved in interaction with β2M (Figure 2, A–C). Since the C-terminal end of ESAT-6 in the ESAT-6:CFP-10 complex is essentially exposed [14] (Figure S3), it is possible that ESAT-6 while in complex with CFP-10 can still interact with β2M. Therefore, the ability of the ESAT-6:CFP-10 complex to interact with β2M was examined by a co-immunoprecipitation assay. Affinity purified His-tagged ESAT-6:CFP-10 or ESAT-6ΔC:CFP-10 or CFP-10 alone was incubated with THP-1 macrophage lysate. Mouse anti-human β2M Ab was then added to these mixtures and detection of β2M-bound His-tagged ESAT-6 protein was carried out using rabbit anti-His Ab. We were able to detect a band corresponding to the size of His-tagged ESAT-6 in the reaction mixture containing ESAT-6:CFP-10 (Figure 2D, Lane 6), however, ESAT-6ΔC:CFP-10 or CFP-10 does not interact with β2M present in the THP-1 extract (Figure 2D, Lanes 7 and 8). Similarly, ESAT-6:CFP-10 was also found to interact with mouse β2M (Figure S4). These results suggest that ESAT-6 alone or in complex with CFP-10 is capable of interacting with β2M and the C-terminal six amino acid residues (90–95) of ESAT-6 are crucial for this interaction.

So far we observed that the C-terminal end of ESAT-6 was involved in interaction with β2M. Since β2M is an integral part of the functional MHC class I molecules, we next examined whether ESAT-6 can interact with HLA-bound β2M also. Therefore, PMA-differentiated THP-1 macrophage lysate was mixed and incubated with either recombinant ESAT-6 or ESAT-6:CFP-10 and the interacting complexes were pulled down using mouse anti-human HLA class I and probed with anti-His as well as anti-β2M antibodies. It was found that β2M but not ESAT-6 could be immunoprecipitated along with HLA-I (Figure S5). These results indicate that ESAT-6 binds only to the free β2M, but not to that already in complex with HLA-I heavy chain.

### ESAT-6:β2M interaction is not affected by high salt concentrations and pH

**Figure 3:**
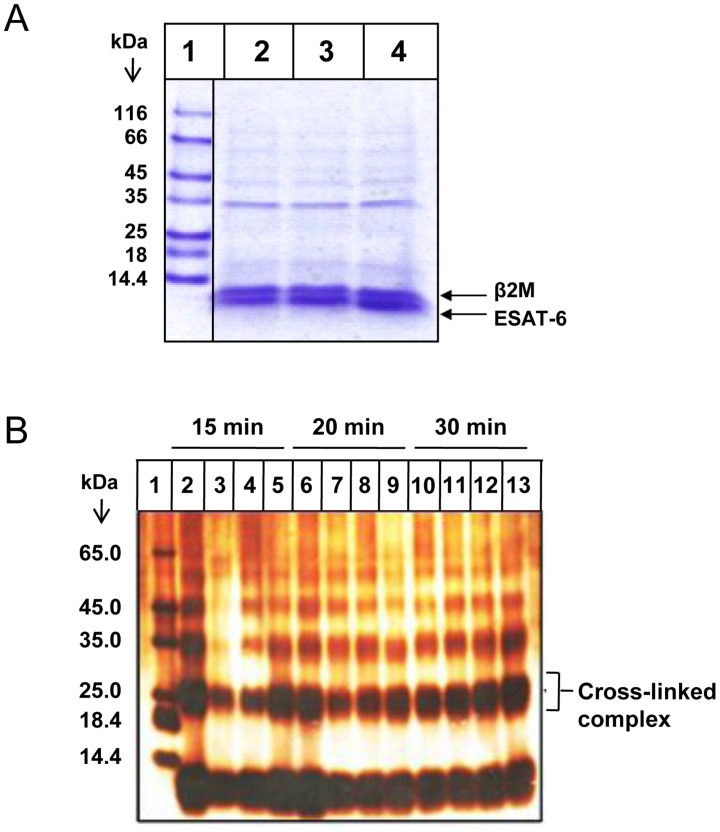
The ESAT-6:β2M complex is stable at high salt concentrations and low pH. (A) Cobalt agarose bead-bound ESAT-6:β2M complex was washed with increasing concentration of 0.5 M NaCl (Lane 2), 1 M Nacl (Lane 3) and 2 M NaCl (Lane 4). Proteins bound to these beads were eluted by boiling in 1× PAGE loading dye and resolved on a 16% Tris-Tricine SDS-PAGE gel and visualized by Coomassie blue staining. Lane 1 shows the molecular weight marker. (B) Purified ESAT-6:β2M protein complex was dialyzed in acetate buffers of varying pH like pH 4.0 (Lanes, 2, 6 and 10), pH 5.0 (Lanes, 3, 7 and 11), pH 6 (Lanes, 4, 8 and 12) and pH 8.0 (Lanes, 5, 9 and 13). After dialysis, the protein complexes were cross-linked by glutaraldehyde treatment for 15, 20 and 30 minutes at 37°C and resolved on a 16% Tris-Tricine SDS-PAGE gel and visualized by silver staining. Protein molecular weight marker was loaded in Lane 1. Results are representative of three different experiments.

Interestingly, the interaction between ESAT-6 and β2M was found to be significantly stable at high ionic concentrations. The β2M in the ESAT-6:β2M complex immobilized to talon beads failed to dissociate from the His-tagged ESAT-6 protein even after washing with buffers containing NaCl as high as 2 M (Figure 3A). Stability of the ESAT-6:β2M complex in the presence of high salt and the neutral nature of the amino-acids indicate that the ESAT-6:β2M complex is stabilized by hydrophobic rather than ionic interactions.

By changing the protonation state of the charged residues, the pH can significantly influence the nature of protein-protein interactions. pH can considerably dictate the strength and geometry of electrostatic interactions, conformation of the interacting proteins, as well as protein hydration which are critical in stabilization of protein-protein interactions. Therefore, the effect of pH was examined on the stability of ESAT-6:β2M complex. The stability of the ESAT-6:β2M complex at various pH was confirmed by glutaraldehyde cross-linking of ESAT-6 and β2M. Covalent cross-linking of two proteins is usually considered to be a convincing evidence of their physical proximity and interaction. Glutaraldehyde was found to covalently cross-link ESAT-6 and β2M at pH 4.0, 5.0, 6.0 and 8.0. The covalently cross-linked ESAT-6:β2M protein complex was detectable as a ∼24 kDa band on a denaturing SDS gel (Figure 3B). These observations further confirm the stability of the ESAT-6:β2M complex across a wide pH range.

### ESAT-6 or ESAT-6:CFP-10 is trafficked into the ER

The functional implications of protein-protein interactions depend on the spatial and temporal localization of the interacting partners within the cell. Therefore, in order to have a pathophysiological role in the host-pathogen interaction, ESAT-6 and/or ESAT-6:CFP-10 must find its way to the cellular compartments where β2M is present. β2M is known to be present in high concentration within endoplasmic reticulum (ER) where it is synthesized and undergoes necessary post-translational modifications and associates with the alpha chain of the MHC-I, as well as other class I like molecules like CD1 and hemochromatosis protein (HFE) that are transported to the cell surface via the golgi apparatus [23], [24].

**Figure 4:**
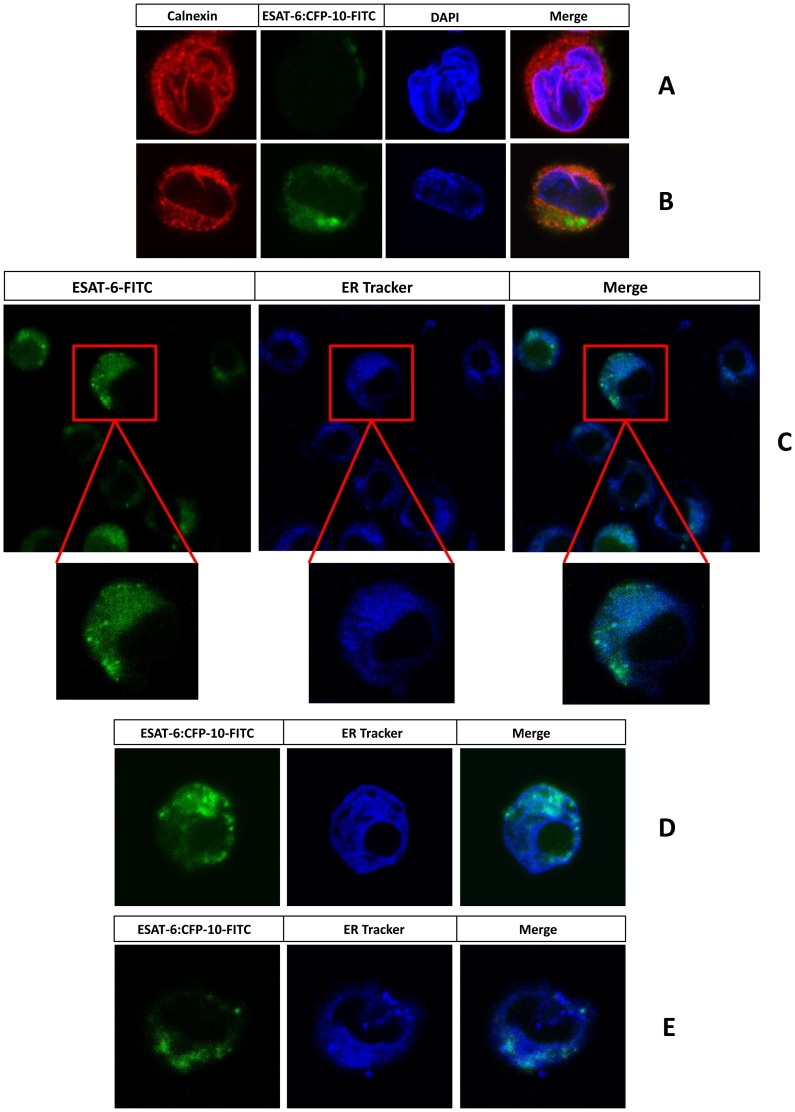
Exogenously added ESAT-6 or ESAT-6:CFP-10 complex can enter into ER. PMA-differentiated THP-1 macrophages were incubated with FITC-labelled ESAT-6:CFP-10 (green) either at 4°C (A) or 37°C (B) for about 120 minutes. Cells were then washed, fixed, permeabilised and stained for an ER marker calnexin using rabbit anti-calnexin Ab and Alexa Fluor 594 conjugated anti-rabbit secondary Ab (red). In another set of experiments KG-1 dendritic like cells (C) or PMA-differentiated THP-1 macrophages (D) or thioglycolate-elicited C57BL/6 mouse peritoneal macrophages (E) were treated with FITC-labelled ESAT-6 or ESAT-6:CFP-10 (green) for about 100 minutes at 37°C and then incubated with ER-Tracker dye (blue) for another 20 minutes and observed under the LSM 510 Meta confocal microscope. Results are representative of three different experiments.

Interestingly, ESAT-6 and/or ESAT-6:CFP-10 complex secreted by the *M. tuberculosis* bacilli in the phagosome or cytoplasm can make its way out by lysing the cell membranes [25], [26] which can be taken up by the surrounding macrophages and dendritic cells. Another possible source of soluble ESAT-6 and/or ESAT-6:CFP-10 is mycobacteria surviving in the extracellular milieu of caseating granulomas where the proteins secreted by the extracellular bacilli are available for uptake by antigen presenting cells present in these granulomas [27]. Interestingly, studies have indicated that soluble proteins can gain direct access to the ER lumen of dendritic cells [28]. Therefore, soluble ESAT-6 and/or ESAT-6:CFP-10 taken up by macrophages and dendritic cells may be retrotranslocated directly to the ER of these cells which would enable it to interact with β2M and modulate β2M-dependent responses. To test this, THP-1 macrophages were incubated for 2 hours with FITC-labelled ESAT-6 or ESAT-6:CFP-10 and their localization was tracked along with the ER-specific marker calnexin by confocal microscopy. The ESAT-6:CFP-10 was found to be present in the ER (Figure 4B). Similarly, FITC-labelled ESAT-6:CFP-10 complex was also found to be present within the ER-tracker dye-positive regions in THP-1 macrophages (Figure 4D) as well as in mouse peritoneal macrophages (Figure 4E). Similarly, FITC-labelled ESAT-6 alone was found to be present in the ER-tracker dye-positive regions of KG-1 dendritic like cells (Figure 4C). FITC-labelled ESAT-6:CFP-10 was incubated with THP-1 macrophages at 4°C as a negative control for internalisation (Figure 4A). These observations suggest that soluble ESAT-6 and ESAT-6:CFP-10 can translocate into the ER lumen from the extracellular milieu to interact with the resident β2M in KG-1 cells and macrophages. From the confocal microscopy data, it appears that ESAT-6:CFP-10 is able to enter the vesicles of the phagocytic pathway as well as other cellular compartments since all the FITC signal emanating from labelled ESAT-6:CFP-10 did not superimpose with the signal emanating from ER-specific markers (Figure 4). However, as is evident from the data, sufficient quantities of ESAT-6/ESAT-6:CFP-10 are able to translocate to the ER where the complex is available for possible interaction with β2M.

### ESAT-6 or ESAT-6:CFP-10 inhibits surface expression of β2M

**Figure 5:**
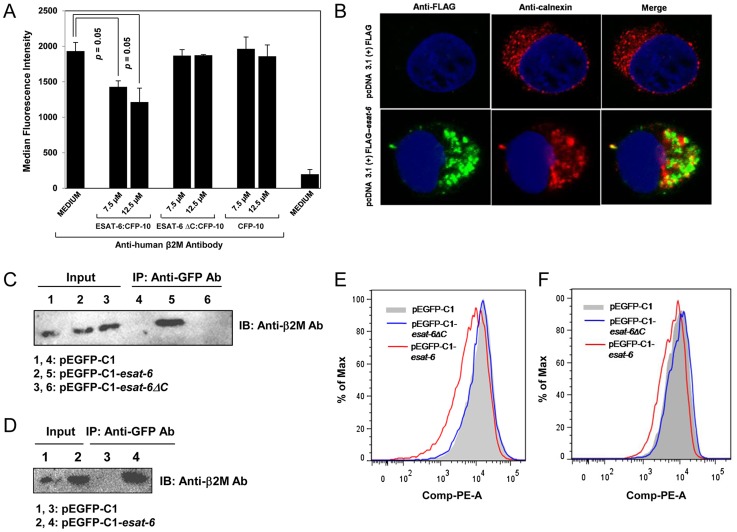
Exogenous addition of ESAT-6:CFP-10 complex or transient expression of ESAT-6 downregulates expression of surface β2M. (A) PMA-differentiated THP-1 macrophages were treated with ESAT-6:CFP-10 or ESAT-6ΔC:CFP-10 or CFP-10 protein for 2 hours and stained with PE conjugated anti-human β2M Ab for measuring surface expression of β2M by flow cytometry. Median fluorescence intensities of the β2M levels of various groups were calculated and the results are shown as mean ± SD of 3 different experiments. (B) HEK-293 cells were transfected with either pcDNA 3.1 (+)-FLAG control plasmid or pcDNA 3.1 (+)-FLAG-*esat-6* and after 20–24 hours, cells were fixed, permeabilized and stained with anti-calnexin Ab followed by Alexa Fluor 594 conjugated anti-rabbit secondary Ab (red) to visualize the endoplasmic reticulum and anti-FLAG Ab followed by Alexa Fluor 488 conjugated secondary anti-mouse Ab (green) to visualize intracellular ESAT-6. Nucleus was visualized by DAPI staining (blue). Cells were observed under confocal microscope. (C) Lysates prepared from the HEK-293 cells transfected with either pEGFP-C1 or pEGFP-C1-*esat-6* or pEGFP-CI-*esat-6ΔC* were incubated with anti-GFP Ab bound to protein A/G agarose beads. Immunoprecipitated complexes (Lanes 4–6) were separated on a 15% SDS-PAGE and transferred to a nitrocellulose membrane which was probed with anti-β2M Ab. About 10% of the lysates were loaded as input control (Lanes 1–3). (D) HEK-293 cells were transfected with either pEGFP-C1 or pEGFP-C1-*esat-6* and after 20–24 hours, cells were used to prepare enriched rough endoplasmic reticulum fractions (RER). Equal amounts of proteins extracted from the enriched RER fractions were incubated with anti-GFP Ab bound to protein A/G agarose beads (Lanes 3 and 4). Immunoprecipitated complexes were separated on a 15% SDS-PAGE and transferred to a nitrocellulose membrane and was probed with anti-β2M Ab. About 10% of the lysates of enriched RER fractions (Lanes 1 and 2) were loaded as input controls. (E) THP-1 cells were nucleofected and (F) HEK-293 cells were transfected with either pEGFP-C1 or pEGFP-C1-*esat-6* or pEGFP-CI-*esat-6ΔC* and after 20–24 hours, cells were stained with PE conjugated anti-human β2M Ab and β2M expression on the cell surface was measured by flow cytometry for EGFP-positive cells. Results are representative of three different experiments.

In the ER lumen, β2M associates with MHC class I molecules to form class I complexes which are then loaded with peptide and transported to the cell surface for antigen presentation to CD8^+^ T cells. It is possible that once translocated to the ER, ESAT-6:CFP-10 can interact and sequester β2M and thereby reduce the availability of free β2M to form complex with HLA class I molecules. In such situations, the surface levels of both MHC and β2M molecules are likely to be decreased. Expectedly, when THP-1 macrophages were incubated with recombinant ESAT-6:CFP-10 protein at two different concentrations [17], [25], surface β2M levels were found to be decreased in a concentration dependent manner (Figure 5A and Figure S6). Interestingly, no significant changes in the surface expression of β2M were noticed in the presence of mutant ESAT-6ΔC:CFP-10 complex or CFP-10 alone (Figure 5A), indicating that suppression of β2M surface expression was possibly due to its sequestration by the intact C-terminal region of ESAT-6. To rule out the possibility that the reduction in surface β2M levels was due to toxicity of the ESAT-6:CFP-10 complex, an MTT viability assay was performed with THP-1 macrophages treated with ESAT-6:CFP-10. We found that the viability of the ESAT-6:CFP-10 treated cells was not affected (Figure S7). Another possibility is that incubation with ESAT-6:CFP-10 may have caused general trafficking defects to reduce the surface expression of β2M. To rule out this, we examined the surface expression of other cell surface markers like Mac-1, TLR4, MHC-II and CD14 using flow cytometry. Levels of all these molecules were found to remain unchanged in ESAT-6:CFP-10 treated cells (Figure S8). ESAT-6:CFP-10 also did not affect β2M expression at the protein and mRNA level (Figure S9). These data indicate that the reduction of surface β2M levels was possibly due to physical sequestration of β2M by ESAT-6 inside the ER. *M. tuberculosis* may escape from the phagosome to the host cytoplasm of infected cells in an RD1 dependent manner and secrete ESAT-6:CFP-10 directly into the cytoplasm of infected cells [26] which may find its way into the ER lumen to interact with the resident β2M. To test this hypothesis, we over-expressed FLAG- or GFP-tagged ESAT-6 in cells in an attempt to mimic physiological conditions where ESAT-6 is secreted directly into the cytosol. We transfected HEK-293 cells with pcDNA 3.1(+)-FLAG-*esat-6* and studied localization of ESAT-6 (stained with anti-FLAG Ab) in ER by confocal microscopy and was found to be present in the calnexin-positive ER compartments (Figure 5B) suggesting that intracellular ESAT-6 can also find its way into the ER. We also performed co-immunoprecipitation studies in cells over-expressing full length ESAT-6 or ESAT-6ΔC cloned with an N-terminal EGFP tag in pEGFP-C1. ESAT-6 and β2M complexes could be pulled down from the whole cell lysates of HEK-293 cells over-expressing pEGFP-C1-*esat-6* but not from cells transfected with pEGFP-C1 vector alone or pEGFP-C1-*esat-6ΔC* (Figure 5C). Also, we were able to pull down the ESAT-6:β2M complex from the enriched ER fraction of HEK-293 cells transfected with pEGFP-C1-*esat-6* but not from cells transfected with the vector alone, indicating that ESAT-6:β2M complex was present inside the ER (Figure 5D). These data suggest that intracellular ESAT-6 is not only able to enter the ER but also can interact with the ER-resident β2M. We next examined, whether such interaction of ESAT-6 with β2M in ER has any effect on the surface β2M levels. Therefore, THP-1 cells were nucleofected and HEK-293 cells were transfected with pEGFP-C1, pEGFP-C1-*esat-6* or pEGFP-C1-*esat-6ΔC*. Nucleofection efficiency for THP-1 was found to be ∼30% and transfection efficiency for HEK-293 was found to be ∼50%. Surface β2M expression on GFP positive cells was measured by flow cytometry using PE conjugated anti-human β2M Ab. It was observed that the intracellular expression of ESAT-6 resulted in reduction of surface β2M levels in THP-1 (Figure 5E) and HEK-293 (Figure 5F) cells when compared to the cells transfected with pEGFP-C1 or pEGFP-*esat-6ΔC*.

### ESAT-6:CFP-10 treatment affects expression of HLA class I molecules

**Figure 6:**
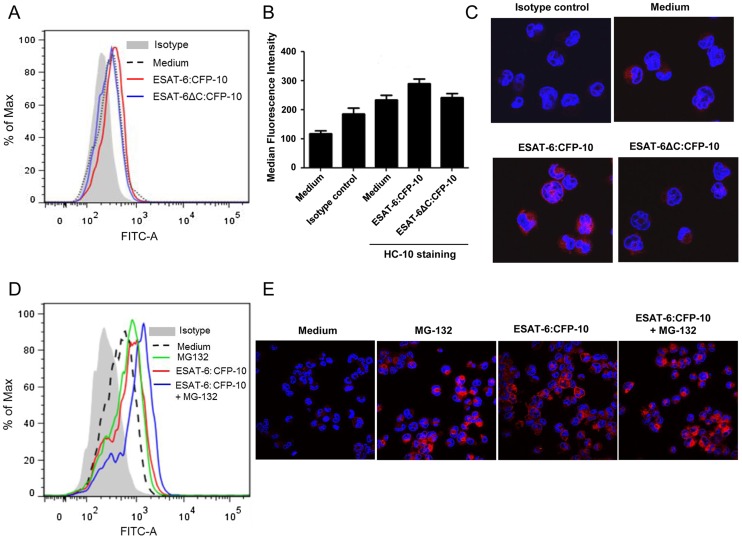
Soluble ESAT-6:CFP-10 increases the levels of free HLA class I heavy chain molecules. (A) PMA-differentiated THP-1 macrophages were treated with either ESAT-6:CFP-10 or ESAT-6ΔC:CFP-10 protein (12.5 µM each). After 2 hours, cells were washed and incubated with anti-HLA class I heavy chain mAb HC-10 followed by FITC conjugated anti-mouse secondary Ab. Surface expression of free HLA class I heavy chain molecules were studied by flow cytometry. Cells stained with appropriate isotype Ab were used as control. (B) Median fluorescence intensities of different experimental groups described in Figure 6A were calculated and the results are shown as mean ± SD of 3 different experiments. (C) THP-1 macrophages pre-treated with either ESAT-6:CFP-10 or ESAT-6ΔC:CFP-10 protein (12.5 µM each) were fixed, permeabilized and stained with HC-10 Ab followed by Alexa Fluor 594 conjugated anti-mouse secondary Ab (red). Matching isotype Ab was used as control. The nucleus was visualized by DAPI staining (blue). The stained cells were observed under a confocal microscope. (D) In another set of experiments, THP-1 macrophages were pre-treated for 30 minutes with 5 µM MG-132 followed by incubation with either ESAT-6:CFP-10 or ESAT-6ΔC:CFP-10 protein (12.5 µM each) for 2 hours. Free HLA class I molecules on the cell surface were stained with HC-10 Ab followed by staining with FITC conjugated anti-mouse secondary Ab and studied by flow cytometry. Isotype-matched Ab was used as control. (E) THP-1 macrophages treated with either ESAT-6:CFP-10 or ESAT-6ΔC:CFP-10 protein (12.5 µM each) in the absence or presence MG-132 werefixed, permeabilized and stained with HC-10 Ab followed by Alexa Fluor 594 conjugated secondary anti-mouse Ab (red). Nucleus was stained with DAPI (blue) and cells were visualized under a confocal microscope. Data shown is representative of three independent experiments.

Beta-2-microglobulin remains associated with HLA class I, CD1 and HFE molecules on the cell surface. Any change in the levels of β2M would probably lead to changes in the cell surface expression of HLA class I, CD1 and HFE. As ESAT-6:CFP-10 treatment leads to reduction in surface expression of β2M (Figure 5), we next examined whether it has any effect on HLA class I expression. For this purpose, we utilized two different types of anti-HLA antibodies: HC-10 and W6/32. HC-10 is a monoclonal Ab that can detect only the free HLA-I heavy chain molecules not complexed with β2M [29]. Newly synthesized HLA molecules form a tri-molecular complex with β2M and antigenic peptide which is transported to the surface to present the peptide to its cognate T cell receptor (TCR) [30]. In contrast, it has been reported that heavy chains of MHC class I alleles can also progress to the cell surface alone without being associated with β2M. Such free MHC class I heavy chains are present normally on cells, especially in activated lymphocytes [31], dendritic cells and macrophages and have been found to be upregulated in patients suffering from spondyloarthropathy [32]. Interestingly, we observed an increased binding of HC-10 Ab in THP-1 macrophages treated with ESAT-6:CFP-10 but not with ESAT-6ΔC:CFP-10 (Figures 6A and 6B). Increased HC-10 staining upon addition of ESAT-6:CFP-10 was observed also by confocal microscopy where intracellular levels of the free HLA molecules can also be determined (Figure 6C). These data clearly suggest that the levels of β2M-free HLA class I molecules were increased on the surface as well as intracellularly after treatment with ESAT-6:CFP-10. Free HLA class I molecules are not only transported to the cell surface but a portion may also undergo proteasomal degradation since addition of MG-132 (a proteasomal inhibitor) to THP-1 macrophages resulted in increased HC-10 staining which was further intensified in the presence of ESAT-6:CFP-10 (Figures 6D and 6E). Therefore, it appears that once ESAT-6:CFP-10 complex sequesters β2M inside the ER, the number free HLA class I heavy chain molecules are increased due to less availability of free β2M molecules to associate with them. This would possibly explain the increase in HC-10 staining observed in the presence of ESAT-6:CFP-10.

**Figure 7:**
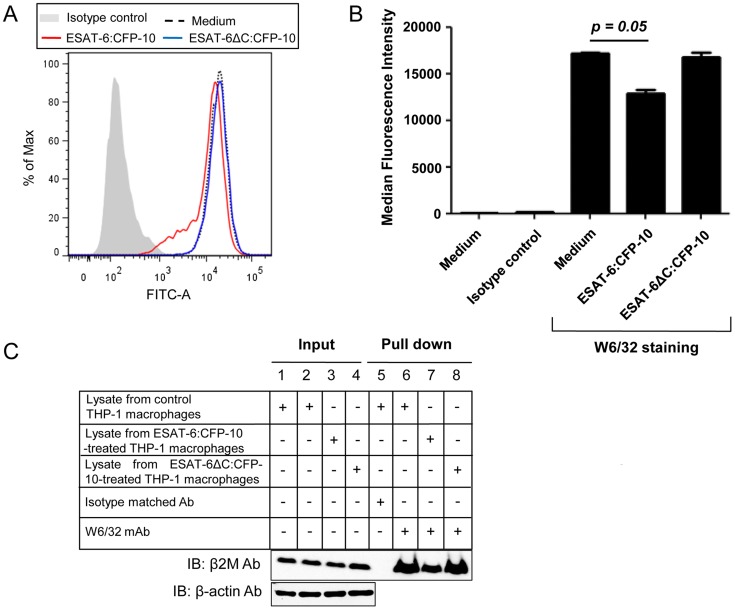
Soluble ESAT-6:CFP-10 reduces surface levels of β2M-associated HLA class I molecules. (A) PMA-differentiated THP-1 macrophages were treated with 12.5 µM of either ESAT-6:CFP-10 or ESAT-6ΔC:CFP-10 protein for 2 hours. Cells were stained with (W6/32) mAb followed by FITC conjugated anti-mouse secondary Ab. Expression of surface β2M conjugated HLA class I molecules was studied by flow cytometry. Isotype-matched Ab was used as control. (B) Median fluorescence intensities of different experimental groups of Figure 7A were calculated and the results are shown as mean ± SD of 3 different experiments. (C) THP-1 macrophages were either left untreated (control) or treated with 12.5 µM of ESAT-6:CFP-10 or ESAT-6ΔC:CFP-10. After 2 hours, cell were harvested and lysates were prepared. Equal amount of protein from each experimental group was incubated with W6/32 mAb bound to protein A/G agarose. Isotype matched Ab was used as control. Pulled-down complexes (Lanes 5–8) were resolved on a 15% glycine SDS-PAGE and transferred onto a nitrocellulose membrane which was probed with anti-β2M Ab. About 10% of the lysate was used as input controls (Lanes 1–4, upper panel). Equal loading in the input samples was also confirmed by probing the input controls with anti-β-actin Ab (Lanes 1–4, lower panel).

On the other hand, when the surface expression of β2M-complexed HLA class I molecules was measured with the help of W6/32, (a monoclonal Ab that recognizes a conformation specific epitope on the HLA class I molecules only when associated with β2M [33], [34]) we observed a significant decrease in staining in ESAT-6:CFP-10-treated but not in ESAT-6ΔC:CFP-10-treated cells (Figures 7A and 7B). Also, a pull down assay with W6/32 yielded lesser amount of β2M in ESAT-6:CFP-10 treated cells, indicating that less amount of β2M was complexed with HLA class I molecules in these cells compared to untreated as well as those treated with ESAT-6ΔC:CFP-10 (Figure 7C). Data from Figures 6 and 7 indicate that ESAT6:CFP-10 can sequester free β2M in ER resulting in reduced HLA class I-β2M complex formation and consequently increasing the levels of free HLA class I heavy chain molecules.

### ESAT-6:CFP-10 inhibits MHC-I presentation of SIINFEKL peptide derived from cytoplasmic and soluble ovalbumin

**Figure 8:**
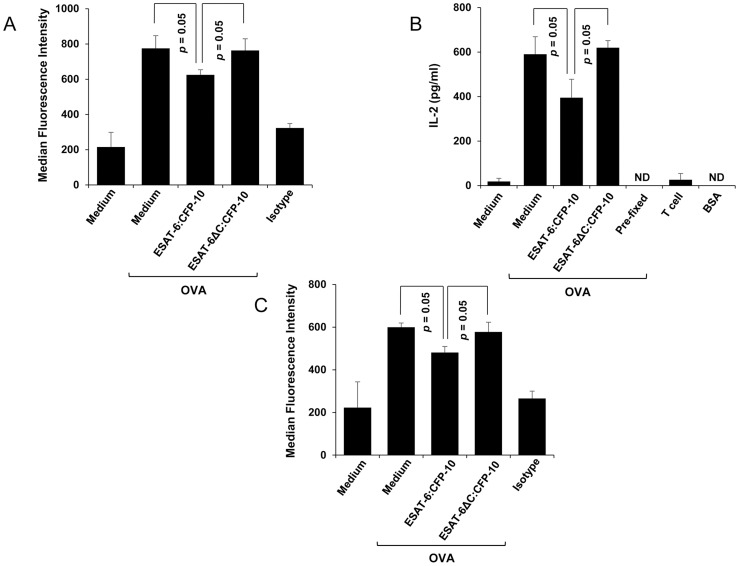
Soluble ESAT-6:CFP-10 complex inhibits presentation of the SIINFEKL peptide. (A) OVA (10 mg/ml) was hypertonically loaded on to C57BL/6 mouse peritoneal macrophages pre-treated with 7.5 µM of ESAT-6/CFP-10 or ESAT-6:CFP-10ΔC. Presentation of SIINFEKL peptide derived from OVA on MHC-I is quantitated by FACS using an Ab that recognizes SIINFEKL bound to H-2K^b^ MHC-I and median fluorescence intensities of SIINFEKL presentation of different groups were calculated and the results are shown as mean ± SD of 3 different experiments. The OVA pulsed cells were stained with PE conjugated isotype-matched control Ab. (B) Responses of the MHC class I-restricted T cell line B3Z to peritoneal macrophages presenting hypertonically loaded cytoplasmic OVA. Pre-fixed macrophages pulsed with OVA antigen and BSA were used as controls. (C) C57BL/6 mouse peritoneal macrophages pre-treated with 7.5 µM of soluble ESAT-6/CFP-10 or ESAT-6:CFP-10ΔC were incubated with 1 mg/ml of exogenously added soluble OVA and analyzed for cross-presentation of SIINFEKL peptide on H-2K^b^ MHC-I by FACS using an Ab that recognises SIINFEKL bound to H-2K^b^ MHC-I. Median fluorescence intensities of SIINFEKL presentation of different groups were calculated and the results are shown as mean ± SD of 3 different experiments.

Most cytoplasmic antigens are processed by the proteasomal machinery and the derived restricted peptides are presented by the MHC-I molecules to CD8^+^ T cells. We have observed earlier that surface expression of β2M and MHC-I molecules is reduced in macrophages treated with ESAT-6:CFP-10 (Figures 5A, 7A and 7B). We also found that ESAT-6:CFP-10 complex is able to enter vesicles of the phagocytic system and ER network (Figure 4 and Figure 5B) and by interacting and sequestering β2M can reduce β2M-MHC-I complex formation (Figure 7C). Therefore, we expected a reduction in the amount of peptides derived from the cytoplasmic antigens to be presented on MHC-I-β2M complex at the cell surface leading to inefficient presentation of antigen-derived peptides to CD8^+^ T cells. Therefore, thioglycolate-elicited mouse peritoneal macrophages from C57BL/6 mice (H-2K^b^) were cytosolically loaded with native ovalbumin (OVA) and the levels of ovalbumin-derived SIINFEKL peptide (OVA_257–264_) presented by H-2K^b^ was examined by flow cytometry in the absence or presence of ESAT-6:CFP-10 or ESAT-6ΔC:CFP-10 complex. Our data indicate that the surface levels of SIINFEKL in complex with H-2K^b^ were significantly reduced in cells treated with ESAT-6:CFP-10 protein when compared with ovalbumin-pulsed cells treated with either medium or ESAT-6ΔC:CFP-10 protein (Figure 8A). We also confirmed the staining experiment with an IL-2 assay as a read out for T cell function. The results show that the decreased SIINFEKL staining in cells treated with ESAT-6:CFP-10 actually correlates with a functional defect in presentation of ovalbumin antigen (Figure 8B).

Cross-presentation is a process by which endocytosed antigens are processed and presented by the MHC-I system. Cross-presentation is thought to be highly relevant to the development of adaptive immunity against *M. tuberculosis* infection [35]. Therefore, presence of ESAT-6 in the ER can also affect cross-presentation of exogenously added antigens. When peritoneal macrophages from C57BL/6 mice were pre-treated with soluble ESAT-6:CFP-10, washed and incubated with soluble ovalbumin antigen, cross-presentation of SIINFEKL peptide derived from the soluble ovalbumin on MHC-I was poor as compared to the cells treated with either medium or ESAT-6ΔC:CFP-10 protein (Figure 8C). This study indicates that ESAT-6:CFP-10 also affects the cross-presentation of endocytosed antigens.

### The ESAT-6:β2M complex exists in the pleural biopsy of individuals suffering from pleural TB

**Figure 9:**
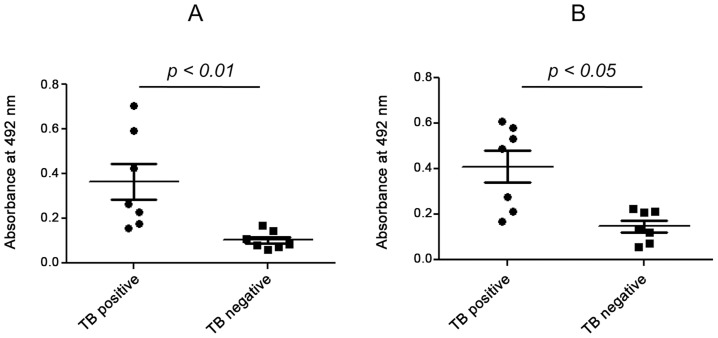
Detection of ESAT-6:β2M complex in pleural fluid. Pleural fluid samples from 7 pleural TB positive and 7 TB negative patients were subjected to sandwich ELISA. The amount of β2M (A) and ESAT-6:β2M complex (B) was measured and compared on the basis of absorbance at 492 nm between TB positive and TB negative patients.

Pleural TB is known to be the second most common form of extra-pulmonary TB. Rupture of caseous granulomas into the pleura marks the beginning of pleural tuberculosis. The conditions manifested in pleural TB resemble those observed in a delayed type hypersensitivity reactions [36]. It has been reported that the pleural fluid in the pleural TB patients contains considerably higher amounts of β2M [37]. ESAT-6 and CFP-10 were also found to be present in the pleural effusions [38]. Indeed, we were able to find significantly higher levels of β2M in the pleural biopsy of the pleural TB patients as compared to those suffering from non-TB pleuritis (Figure 9A). The presence of ESAT-6:β2M complexes in these samples were detected by using a sandwich enzyme linked immunosorbent assay (ELISA) where anti-β2M Ab was used for capture and anti-ESAT-6 Ab was used for detection. The pleural TB positive samples with higher β2M levels were also found to have higher ESAT-6:β2M complexes compared to the patients suffering from non-tuberculous pleuritis (Figure 9B). Results of the sandwich ELISA indicate that the ESAT-6:β2M complex can also exist in pathophysiological settings like pleural fluid of individuals suffering from pleural TB.

## Discussion

ESAT-6 and CFP-10 of *M. tuberculosis* are secreted by the ESX-1 system into the host and believed to contribute to mycobacterial virulence [20]. However, the exact molecular mechanism by which ESAT-6 exerts its virulence effects is not well understood. Using a yeast two hybrid screening, where ESAT-6 was used as bait to screen a human leukocyte prey cDNA library, we were able to identify β2M as an interacting partner of ESAT-6. The yeast two-hybrid data for ESAT-6:β2M interaction was further confirmed by surface plasmon resonance analysis, co-purification, GST pull down and co-immunoprecipitation assays. The data from all these studies point to a strong interaction between ESAT-6 and β2M. The ESAT-6:β2M interaction was found to be stable across a wide pH range and salt concentrations as high as 2M indicating interaction between these two proteins is stabilized by hydrophobic interactions.

β2M is a ∼13 kDa polypeptide that is found in the serum as well as in tight association with MHC-I heavy chain on the surface of nearly all nucleated cells [39]. β2M is also associated with CD1, involved in the presentation of lipid antigens to T cells [40] and HFE which is known to regulate intracellular iron concentration [41]. Association of β2M with the α chain of HLA/MHC-I, CD1 and HFE is a prerequisite for the cell-surface expression of these receptors [39]–[42]. We observed that over-expression of ESAT-6 in the cell cytoplasm or exogenous addition of ESAT-6:CFP-10 complex reduced the cell surface translocation of β2M in macrophages without affecting the total cellular β2M content. This may potentially lead to interference not only with protein and lipid antigen presentation but also with iron metabolism. Iron is essential for the growth of *M. tuberculosis* and therefore manipulation of the host iron homeostasis can be an important virulence strategy for *M. tuberculosis*. Interestingly, HFE knock-out as well as β2M knock-out mice showed an increased susceptibility to experimental infection with *M. avium* and during infection these animals were found to accumulate iron inside the granuloma macrophages [21], [43]. Thus, ESAT-6:CFP-10 perhaps play multiple roles in *M. tuberculosis* virulence.

The dissociation constant of β2M and HLA-AB heavy chain is 1×10^−8^ M [44] and that for β2M and ESAT-6 is 1.03×10^−6^ M, as calculated from the SPR studies. Since, the affinity of β2M for HLA-AB heavy chain is higher than its affinity for ESAT-6, it is most likely that ESAT-6 when present in high concentrations, binds to portion of available free β2M pool before it forms complex with the HLA heavy chain. This probably explains the partial inhibition observed in the surface expression of β2M and HLA class I and class I-restricted antigen presentation. Overall, our results suggest that ESAT-6 if present in the cell at a given point in time, as can happen during the course of infection, can potentially compromise the function of β2M, HLA class I molecules and interfere with antigen presentation.

Interestingly, UL18, a human cytomegalovirus protein is known to bind β2M and reduce the maturation of cellular MHC-I molecules on cell surface [45]. Also, β2M-deficient mice are found to be more susceptible to mycobacterial infection as compared to the wild-type mice [21], suggesting that β2M and/or β2M-dependent factors are probably important for resistance against mycobacteria. Therefore, it appears logical that targeting β2M and cellular processes regulated by it would be beneficial for the survival of *M. tuberculosis*.

The structural core of the ESAT-6:CFP-10 complex consists of two similar helix–turn–helix hairpin structures from each protein, and is stabilized by an extensive hydrophobic contact surface. The alpha helical cores lie anti-parallel to each other to form a four-helix bundle. The extreme C-terminus of ESAT-6 (residues 84–95) was found to be free and was not involved in interaction with CFP-10 [13]–[15]. Interestingly, a recombinant *M. tuberculosis* strain H37Rv EsxAΔ84–95 expressing a mutant form of ESAT-6 with a C-terminal deletion of 12 amino acid residues was found to possess a functional ESX-1 system (as it was shown to secrete ESAT-6 and CFP-10 efficiently), but was attenuated in a mouse infection model [13]–[15]. This implies that the C-terminus of ESAT-6 does not play a significant structural role, but is required for *M. tuberculosis* virulence. Interestingly, we found that deletion of the last 6 amino acids (valine, threonine, glycine, methionine, phenylalanine and alanine) from the C-terminal end of ESAT-6 inhibited formation of the ESAT-6:β2M complex. The ESAT-6ΔC:CFP-10 complex which failed to interact with β2M also failed to reduce the surface β2M as well as HLA expression. The loss of β2M binding activity thus may well correlate with the attenuation of strains harbouring the mutant ESAT-6 with the last 12 amino acid deletion in the C-terminus region as reported earlier [13]–[15]. It appears that CFP-10 and ESAT-6 stabilizes each other through extensive hydrophobic interactions spanning their core helix-loop-helix structure, while the free flexible C-terminal end of ESAT-6 is available for interaction with β2M and possibly other host proteins. True to this expectation, we found that anti-β2M Ab could co-immunoprecipitate both the ESAT-6 and ESAT-6:CFP-10 complex but not the C-terminally truncated ESAT-6ΔC alone or in complex with CFP-10. Based on the observations made by us and others [13], it is tempting to speculate that the interaction of ESAT-6 through its free C-terminal end with β2M and possibly other host proteins is required for its virulence properties.

Interestingly, exogenously added ESAT-6 and ESAT-6:CFP-10 complex coupled with FITC was able to find its way to the ER of dendritic cells and macrophages. It is interesting to note that fluid phase proteins like human cytomegalovirus (HCMV) protein US6 and the *Pseudomonas aureginosa* derived Exotoxin A that escape proteolytic degradation can enter into the ER lumen directly [28], [46]. However, the exact mechanism by which ESAT-6/ESAT-6:CFP-10 is retrotransported to the ER is not understood. One of the possibilities could be leaching into the cells due to the pore forming ability of ESAT-6 [25], [47]. In fact, *M. tuberculosis* and *M. leprae* but not *M. bovis* BCG are known to rely on ESAT-6:CFP-10 to escape from the phagolysosome to the cytosol in myeloid cells [26]. Again, ESAT-6 but not CFP-10 was able to hemolyse RBCs by forming pores in their membranes which could be plugged by using the appropriate polethylene glycol [47]. These evidence suggest that ESAT-6 or ESAT-6:CFP-10 complex may gain access to the ER compartment either through pore formation or via the cytosolic detour pathway [26], [48]. Exogenously added soluble ESAT-6:CFP-10 complex was found to reduce the amount of SIINFEKL peptide presented by hypertonically loaded cytoplasmic ovalbumin as well as cross-presented ovalbumin indicating that ESAT-6:CFP-10 can affect class I antigen presentation, possibly by sequestering β2M and thus decreasing available free β2M that can associate with MHC-I heavy chain for its surface localization. Poor loading of antigenic peptides on to the mature MHC-I-β2M complexes may in turn could affect the subsequent priming of CD8^+^ T cells to undermine the anti-mycobacterial immunity.

A CD8^+^ T cell response is perhaps crucial for immunity in already established tuberculosis [49]. *M. tuberculosis*-specific CD8^+^ T cells have been found to be present in granulomas and pleural exudates of TB patients sometimes more in numbers than that of CD4^+^ T cells [50], [51]. Moreover, adoptive transfer of CD8^+^ T cells provides protection in BCG and *M. tuberculosis* challenged mice [52], [53]. Also, mice deficient in β2M and TAP are more susceptible to tuberculosis [21], [54]. CD8 deficient mice are still susceptible to *M. tuberculosis* infection though not to the extent of *β2m* ^−/−^ mice [55], [56]. Given the importance of CD8^+^ T cells and the class I antigen presentation pathway in immunity against *M. tuberculosis*, the ability to downregulate CD8^+^ T cell activation can be highly advantageous to *M. tuberculosis* in its constant tug of war with the host immune system. Since, ESAT-6 is highly expressed especially during the initial stages of infection [35] and is a secretory protein, *M. tuberculosis* may deploy ESAT-6 or ESAT-6:CFP-10 complex to sequester β2M in the cell to suppress presentation of mycobacterial antigens and thereby subvert generation of crucial anti-mycobacterial adaptive immune responses. It is also possible that ESAT-6:CFP-10 from resident *M. tuberculosis*, especially macrophages can interfere with the class I antigen presentation pathway and prevent the clearance of infected cells by the cytolytic CD8^+^ T cells. We have carried out experiments that mimic the presence of ESAT-6 both extracellularly and intracellularly. In both the conditions, we found that the surface β2M levels were reduced suggesting a role of ESAT-6 in interfering with class I antigen presentation.

Interestingly, in addition to β2M alone, the ESAT-6:β2M complex could be detected in the pleural fluid of the patients suffering from pleural tuberculosis indicating the pathophysiological importance of ESAT6:β2M interactions *in vivo*. However, the mechanisms leading to the formation of ESAT-6:β2M complex in the pleural fluid are not clear at present.

In summary, our data demonstrate that ESAT-6 and ESAT-6:CFP-10 complex interacts and sequesters one of the host proteins β2M to inhibit surface expression of β2M-associated molecules and consequently can suppress antigen presentation by MHC-I-β2M complex. The free C-terminal end of ESAT-6 is required for such interaction and possibly virulence. However, the exact molecular mechanisms by which the ESAT-6 or ESAT-6:CFP10 complex gains access to the ER resident β2M is yet to be understood. Results presented in our manuscript not only identify a novel interaction but also highlight a novel mechanism of suppression of MHC-I antigen presentation that might play a crucial role in subverting adaptive immune responses during *M. tuberculosis* infection. Therefore, the identification of ESAT-6 and β2M interaction, not only sheds new light on the host pathogen relationship, but also opens up avenues for development of small molecule inhibitors or vaccine for tuberculosis therapy.

## Materials and Methods

### Yeast two-hybrid interaction screening

Library screening was performed according to the user manual (Yeast Protocols Handbook from Clontech). Yeast cell strain AH109 transformed with the bait plasmid pGBKT7-ESAT-6 was mated with yeast strain Y187 transformed with a human leukocyte cDNA prey library obtained from Clontech (Clontech Laboratories, USA). Tranformants were plated and grown on SD/–Ade/–His/–Leu/–Trp medium (QDO plates) for 5 days at 30°C to screen for ADE2 and HIS3 expression. The colonies those appeared on the QDO plates were sub-streaked five times to dilute non-specific library plasmids. The positive colonies were used for bait plasmid isolation as described by Robzyk and Kassir [57]. Prey cDNA was amplified by PCR using primers encompassing the cDNA insert in pACT2 and sequenced using an automated DNA sequencer. The cDNA sequences were identified using online PSI-BLASTX searches (National Center for Biotechnology Information).

### Cell culture

The human monocyte/macrophage cell line THP-1 was obtained from National Centre for Cell Science (NCCS), Pune, India. The suspension cell line was maintained in RPMI-1640 medium (Hyclone, USA) supplemented with 10% (v/v) heat inactivated FBS (Hyclone, USA), antibiotic-antimycotic (1×, containing Penicillin G, Streptomycin, Amphotericin B), 2 mM L-Glutamine and 10 mM HEPES (All from GIBCO, USA) and maintained at 37°C and 5% CO_2_ in a humidified incubator. The KG-1 cells obtained from National Centre for Cell Science (NCCS), Pune, India were grown in Iscove's Modified Dulbecco's Medium or IMDM (HyClone, USA) supplemented with 20% FBS, 1× antibiotic-antimycotic, 2 mM L-Glutamine and 10 mM HEPES and maintained at 37°C and 5% CO_2_ in a humidified incubator. Similarly, HEK-293 cells were maintained in DMEM (Hyclone, USA) supplemented with 10% (v/v) heat inactivated FBS (Hyclone, USA), antibiotic-antimycotic (1×, containing penicillin G, streptomycin, amphotericin B), 2 mM L-Glutamine and 10 mM HEPES (all from GIBCO, USA) and maintained at 37°C and 5% CO_2_ in a humidified incubator. THP-1 monocytic cells were differentiated into macrophages by incubating cells them with 5 ng/ml of PMA (Sigma-Aldrich, USA) for 12 hours followed by a resting period of 24 hours in medium containing 10% FBS. For some experiments, peritoneal macrophages were harvested from C57BL/6 mice obtained from National Institute of Nutrition (NIN), Hyderabad. Mice of either sex, 6–8 weeks old, were injected intra-peritoneally with 1 ml of 4% thioglycolate broth and after 3 days, peritoneal macrophages were isolated as described earlier [58], [59]. The mice were maintained and the experiments were conducted at the animal house facility of National Institute of Nutrition (NIN), Hyderabad following the guidelines of the Institutional Animal Ethics Committee. Harvested cells were washed and used for further experiments. All cells (THP-1 macrophages and thioglycolate elicited mouse peritoneal cells) were incubated with recombinant proteins in incomplete culture medium without FBS for 2 hours. For some experiments, before the addition of recombinant proteins, cells were treated with 10 µM MG-132 (Calbiochem, USA).

### Purification of recombinant proteins

ESAT-6 ORF cloned in pET23a was used for purification of His-tagged ESAT-6 as described elsewhere [20]. In brief, protein expression was induced in *E. coli* strain BL21 (DE3) transformed with pET23a-ESAT-6 by addition of isopropyl-β-D-galactopyranoside (IPTG) (Sigma-Aldrich, USA) to a final concentration of 1 mM. ESAT-6 protein was purified from cell pellet under denaturing conditions using 8 M urea (Sigma-Aldrich, USA) and the denatured protein was refolded back by sequential removal of urea. For purification of mutant ESAT-6 (ESAT-6ΔC, the last 6 amino acids were deleted from the C-terminal end of ESAT-6) and CFP-10, the corresponding ORFs were cloned in pET23a with an N-terminal His-tag. For purification of the protein complexes like ESAT-6:CFP-10 and ESAT-6ΔC:CFP-10, ESAT-6 or ESAT-6ΔC ORF was cloned in frame with His-tag of first multiple cloning site (MCS) in pETDuet (Novagen EMD, Germany) while CFP-10 was cloned in the second MCS of pETDuet vector. For ESAT-6:β2M purification, ESAT-6 was cloned in frame with His-tag of the first MCS in pETDuet while β2M was cloned in the second MCS of pETDuet vector. These clones were transformed in *E. coli* strain BL21 (DE3) and protein expression was induced by addition of IPTG. Polyhistidine-tagged recombinant protein was purified from these cells using TALON resin (Clontech Laboratories, USA) according to the manufacturer's recommendation for purification of protein under native condition. Tris-Tricine SDS-PAGE analysis of purified proteins revealed an essentially pure homogenous preparation of expected size. The purified proteins were dialyzed against several changes of 1× PBS and protein concentration was estimated using Bicinchoninic acid method (Pierce Chemical Company, USA).

### Co-immunoprecipitation assay

The recombinant His-tagged protein (100 µg) was incubated with whole cell lysate (500 µg) prepared from PMA-differentiated THP-1 macrophages (made in 1× PBS pH 7.4, 10% v/v glycerol, 1% NP-40 and protease inhibitor cocktail, Roche, Germany) overnight at 4°C on a rotating platform. Mouse anti-human β2M mAb (clone CBL306, IgG1 isotype) (Milipore, USA) was then added to this mixture and incubated for another 2 hours at 4°C. Protein A/G Agarose beads (Santa Cruz Biotechnology, USA) were then added to this mixture and further incubated at 4°C for 1 hour. The protein A/G bound immune complexes were collected by centrifugation, washed extensively with 1× PBS, dissociated by boiling in Laemmli buffer and analysed by Western blotting. In some experiments, HLA class I molecules were pulled down from THP-1 macrophages treated for 2 hours with either ESAT-6:CFP-10 or ESAT-6ΔC:CFP-10 complexes. Post-treatment with protein complexes, the cells were washed and lysed in a HEPES-containing immunoprecipitation buffer (50 mM HEPES pH 7.4, 137 mM NaCl, 10% glycerol and protease inhibitor cocktail, Roche, Germany). Equal amount of lysates (100 µg) were incubated with W6/32 mAb (IgG2a isotype) that recognizes HLA class I complexed with β2M (Abcam Inc, USA) or isotype-matched control Ab (Sigma-Aldrich, USA) overnight, followed by capturing with protein A/G agarose beads (Santa Cruz Biotechnology, USA) for 1 hour. After completion of incubation, the beads were washed, and eluted by boiling in 1× Laemmli buffer and were analysed by Western blotting. For pull down assays in transfected cells, 200 µg whole cell lysate (prepared by using HEPES-containing immunoprecipitation buffer) or RER fraction was incubated with anti-GFP Ab (Sigma-Aldrich, USA) overnight. Immune-complexes were pulled down by addition of protein A/G beads for 1 hour. Beads were washed, resuspended and boiled in 1× Laemmli buffer and the pulled-down products were analysed by Western blotting. About 10% of the lysates were used as input controls in all immunoprecipitation experiments.

### Western blotting

Immunoprecipitated complexes and cell lysates were resolved on either 16% Tris-Tricine SDS or 15% glycine SDS-PAGE and then transferred onto nitrocellulose membranes. The blots were probed with the appropriate combination of primary Ab and HRP conjugated secondary Ab (Sigma-Aldrich, USA). Recombinant His-tagged proteins were detected with rabbit anti-His Ab (Abcam Inc, USA), β2M with rabbit anti-β2M Ab (Abcam Inc, USA) and β-actin with mouse anti-β-actin Ab (Santa Cruz Biotechnology, USA). Bound HRP enzyme was detected by chemiluminescence following the manufacturer's protocol (GE Healthcare, UK).

### Surface plasmon resonance

β2M purified from human urine (Sigma-Aldrich, USA) (dialysed against 10 mM acetate buffer, pH 3.5) was coupled to the flow cells of the sensor chip (Carboxy-Methylated Dextran chip (CM5, Biacore AB) using 100 mM N-hydroxysuccinimide and 400 mM N-Ethyl-N′-(dimethylaminopropyl) Carbodiimide, until an appropriate level of coupling (400 resonance units) was achieved. Deactivation after immobilization was carried out using 1 M ethanolamine (pH 8.5). All binding studies were performed in 75 mM phosphate buffered saline, pH 7.5 using a 5 µl/minute flow rate at 25°C. A control flow cell was also prepared under identical conditions in the absence of the protein. Recombinant His-tagged ESAT-6 protein was dialysed against running buffer and injected in different concentrations into the flow cell with immobilized β2M. Changes in the refraction index at the surface due to interactions between the two molecules were detected and recorded as RU (Resonance Units). Curves generated from the RU trace were evaluated using a curve-fitting algorithm, BIAevaluation (Biacore, GE Healthcare).

### Glutaraldehyde cross-linking to confirm protein-protein interaction

For glutaraldehyde cross-linking, purified ESAT-6:β2M protein complexes were dialysed in sodium acetate buffers of varying pH (4.0, 5.0 and 6.0). The dialysed protein samples were then centrifuged at 14000 rpm for 10 minutes and 100 µl of each sample was treated with 5 µl of 3% freshly prepared solution of glutaraldehyde (Sigma-Aldrich, USA) for 15, 20 and 30 minutes at 37°C. Cross-linking reaction was terminated by addition of 10 µl of 1M Tris-HCl (pH 8.0). Cross-linked products were denatured by heating at 95°C with equal volume of 1× Laemmli buffer, centrifuged and resolved on a 16% Tris-Tricine SDS-PAGE gel and visualized by silver staining.

### GST pull down assay

ESAT-6 or ESAT-6ΔC ORF cloned in pGEX-4T-1 or the empty vector pGEX-4T-1 was used to purify GST-ESAT-6 or GST-ESAT-6ΔC or GST alone using glutathione-agarose beads (Clontech Laboratories, USA) following the manufacturer's protocol. GST-ESAT-6 or GST-ESAT-6ΔC or GST bound to Glutathione bead was incubated with GST pre-cleared THP-1 macrophage lysate (made in lysis buffer composed of 1× PBS, 1% Triton X-100, 1 mM PMSF, 1× protease inhibitor cocktail mixture) at 4°C for 2 hours. The beads were washed with lysis buffer and boiled in Laemmli sample buffer. These samples were then separated by 16% Tris-Tricine SDS-PAGE and analyzed by Western blotting using rabbit anti-human β2M Ab and HRP conjugated anti-rabbit secondary Ab and the blots were visualized by chemiluminescence.

### Detection of ESAT-6:β2M complex in pleural fluid samples from tuberculosis positive individuals

Pleural fluid samples were collected from individuals suffering from clinically diagnosed pleural TB. Patients with non-tuberculous etiology having malignant pleural effusion/parapneumonic effusion were included as controls. The study was approved by the Institutional Ethical Committee (LEPRA Society-Blue Peter Research Centre, Hyderabad) and informed consent was obtained from each subject prior to sample collection. The amount of ESAT-6:β2M complex present in the pleural fluid was measured by sandwich ELISA. Briefly, 96-well ELISA plates were coated overnight with 1 µg/well of mouse anti-human β2M Ab, clone CBL306 (Millipore, USA). After blocking with 2% BSA (AMRESCO, USA), pleural fluid samples (50 µl) were added and incubated overnight at 4°C. Captured ESAT-6:β2M complexes were detected with polyclonal rabbit anti-ESAT-6 Ab (Abcam Inc, USA), diluted 1∶100 and HRP conjugated anti-rabbit Ab (Sigma-Aldrich, USA), diluted 1∶1000. A similar sandwich ELISA was used for detection of β2M in the pleural fluid samples where the ELISA plate was coated with 1 µg/well mouse anti-human β2M Ab, clone CBL306 (Millipore, USA) and post blocking pleural fluid samples (50 µl) were added and the captured β2M was detected with the help of rabbit-anti-human β2M Ab (Abcam Inc, USA), diluted 1∶1000 and HRP conjugated anti-rabbit Ab (Sigma-Aldrich, USA), diluted 1∶1000. All Ab dilutions were made in blocking buffer (1× PBS+2% BSA). To detect the bound HRP, ortho-phenylenediamine or OPD (Sigma-Aldrich, USA) and H_2_O_2_ (Qualigens Fine Chemicals, India) was used. The reaction was stopped by addition of 1N H_2_SO_4_ (Qualigens Fine Chemicals, India). The absorbance was measured at 492 nm. At each step ELISA plates were thoroughly washed 3 times with 1× PBS+0.05% Tween 20 (AMRESCO, USA) and 2 times with 1× PBS.

### Flow cytometry

For flow cytometry, cells were incubated with appropriate antibodies (either as a fluorochrome conjugated primary Ab or in successive steps of unlabelled primary Ab and fluorochrome conjugated secondary reagents) in a 100 µl reaction volume FACS staining buffer (1× PBS containing 1% BSA and 0.1% sodium azide) for 60 minutes on ice. After washing, the cells were fixed in 1% paraformaldehyde. Surface β2M was stained with PE conjugated anti-human β2M Ab of IgM isotype, clone TÜ99 (BD Pharmingen, USA) and PE conjugated mouse IgM, κ (BD Pharmingen, USA) was used as isotype control. Surface-expressed HLA class I molecules were stained with monoclonal antibody HC-10 (kind gift from Prof. H. L. Ploegh) which detects free HLA class I heavy chain molecules or W6/32 (Abcam Inc, USA) which detects β2M complexed HLA class I and FITC conjugated anti-mouse Ab (Sigma-Aldrich, USA). Appropriate isotype-matched IgG2a (Sigma-Aldrich, USA) was used as control. Cell-bound fluorescence was measured on a FACSAria flow cytometer (Beckton Dickinson, USA) and the data were analyzed using FlowJo (TreeStar, Oregon, USA) data analysis software.

### Nucleofection of THP-1 cells

Open reading frame of either full length ESAT-6 or ESAT-6 truncated by 6 C-terminal amino acids residues (ESAT-6ΔC) was cloned in frame with the EGFP protein of pEGFP-C1 (Clontech Laboratories, USA). These constructs, pEGFPC1-*esat-6* and pEGFPC1-*esat-6ΔC*, were nucleofected into 1–2×10^6^ THP-1 cells for cytoplasmic expression of EGFP-ESAT-6 and EGFP-ESAT-6ΔC respectively, using a nucleofection kit from Lonza (Lonza, Germany) following the protocols provided by the manufacturer. Cells nucleofected with empty pEGFP-C1 vector was used as control group. After 20–24 hours, β2M expression on the cell surface was examined by flow cytometry in EGFP-positive cells using PE conjugated anti-human β2M Ab (BD Pharmingen, USA).

### Transfection of HEK-293 cells

ESAT-6 and ESAT-6ΔC ORFs cloned with an N-terminal GFP-tag in pEGFP-C1 vector, pEGFPC1-*esat-6* and pEGFPC1-*esat-6ΔC* respectively, were transfected in HEK-293 cells. For some experiments, ESAT-6 cloned in a pcDNA 3.1 (+) vector with an incorporated N-terminal FLAG tag (cloned between *Nhe*I and *Hind*III restriction sites) was used for transfecting HEK-293 cells. The cells were seeded in antibiotic free DMEM with 10% FCS overnight. Respective plasmids and Lipofectamine 2000 transfection reagent (Invitrogen, USA) were diluted in Opti-MEM minimal serum medium and then mixed together. For 1 µg plasmid 3 µl of Lipofectamine 2000 reagent was used. The plasmid-Lipofectamine mixture was incubated at room temperature for 30 minutes and was added to the seeded HEK-293 cells drop by drop. Cells were kept in Opti-MEM medium for 4–6 hours after which it was replaced with complete DMEM medium. All the experiments were carried out at approximately 20–24 hours post-transfection.

### Confocal microscopy

FITC-labelled ESAT-6:CFP-10 was prepared using a FITC labelling kit from Pierce (Thermo Fisher Scientific, USA) following the manufacturer's protocol. For ER localization studies, one million cells were incubated with FITC-labeled ESAT-6:CFP-10 protein for about 100 minutes and the ER-Tracker dye Blue White DPX (Invitrogen, USA) was added to these cells to a final concentration of 1 µM and incubated for 20 minutes. Cells were washed three times in 1× PBS and then fixed with 3% paraformaldehyde for 10 minutes and mounted on coverslip and visualized under a confocal microscope. Localization of recombinant FITC-labelled ESAT-6:CFP-10 was also studied with calnexin, an ER specific marker. Calnexin was stained with anti-calnexin Ab (Santa Cruz Biotechnology, USA) and detected by Alexa-Fluor 594 conjugated anti-rabbit secondary Ab (Invitrogen, USA). Levels of intracellular free HLA class I molecules not complexed with β2M were measured with a monoclonal Ab HC-10 that specifically detects free HLA class I heavy chain molecules (kind gift from Prof. H.L Ploegh). Alexa Fluor 594 conjugated anti-mouse Ab (Invitrogen, USA) was used as secondary Ab. Intracellularly over-expressed FLAG-tagged ESAT-6 was stained with anti-FLAG Ab (Sigma-Aldrich, USA) followed by Alexa Fluor 488 conjugated anti-mouse Ab. For all experiments involving intracellular staining, cells were first fixed with 3% paraformaldehyde for 10 minutes and then permeabilized with 0.1% Triton X-100 (Sigma-Aldrich) followed by incubation with appropriate primary and secondary antibodies. All cells were visualized under a LSM 510META confocal microscope and images were captured.

### Isolation of rough endoplasmic reticulum (RER) fraction

About 10–15×10^6^ HEK-293 cells were transfected with either pEGFP-C1 or pEGFPC1-*esat-6* or pEGFPC1-*esat-6ΔC* plasmids and after 20–24 hours of transfection, cells were detached, pelleted and washed with 1× PBS. The pellet was used for isolation of the RER fraction using a commercially available kit (IMGENEX, India). Briefly, the cell pellet was resuspended in an iso-osmotic homogenization buffer containing protease inhibitor cocktail (Roche, Germany) and lysed using a Dounce homogenizer. In case of incomplete lysis, cells were also briefly sonicated. Unlysed cells were separated by centrifugation at 1,000 g for 10 minutes. The supernatant from this step was subjected to further centrifugation at 12,000 g for 10 minutes to pellet down mitochondria and cell debris. The supernatant was used for precipitation of RER fraction with 8 mM calcium chloride which was added drop by drop with constant agitation. The RER fraction was pelleted by centrifugation at 8,000 g for 10 minutes at 4°C. The pellet was dissolved in a HEPES containing suspension buffer with protease inhibitor and 1% NP-40 (Sigma-Aldrich, USA). Enrichment of the ER fraction was confirmed by the presence of ER-specific marker β2M (23, 24) and absence of EEA1 (endosome marker), LAMP2 (lysosome marker) as well as GAPDH (cytoplasmic marker) (Figure S10). The enriched ER fraction was stored at −80°C until used for pull-down assays.

### Assay for effect of ESAT-6:CFP-10 complex on MHC-I antigen presentation

Hypertonic cytoplasmic loading of ovalbumin (OVA) followed by osmotic lysis of pinosomes containing OVA was used to present soluble antigens to the MHC-I pathway of antigen presentation [60], [61]. In brief, peritoneal macrophages from C57BL/6 (H-2K^b^) mice were pre-treated with recombinant ESAT-6:CFP-10 or ESAT-6:CFP-10ΔC protein complex (7.5 µM) for 2 hours. After washing, the cells were further incubated for 10 minutes at 37°C in hypertonic serum-free DMEM containing 0.5 M sucrose (Sigma-Aldrich, USA), 10% polyethylene glycol 1000 (Sigma-Aldrich, USA), 10 mM HEPES and 10 mg/ml OVA which is equivalent to 225 mM concentration (Sigma-Aldrich, USA). Then hypotonic DMEM (60% DMEM and 40% water) was added for 2 minutes at 37°C. The cells were washed and further incubated for 2 hours to allow OVA antigen processing and presentation. These cells were then directly probed for the presence of OVA-derived SIINFEKL peptide (OVA_257–264_) on MHC-I using a phycoerythrin (PE) conjugated monoclonal Ab (25-D1.16) that recognizes SIINFEKL peptide bound to MHC-I molecules (H-2K^b^) (eBiosciences, USA). The PE fluorescence was detected in the FL-2 channel of flow cytometer (BD FACSAria) and the data were analyzed using Flowjo software. To determine the effect of soluble ESAT-6:CFP-10 or ESAT-6:CFP-10ΔC protein on cross-presentation of exogenous OVA, peritoneal macrophages from C57BL/6 (H-2K^b^) mice were pre-treated for 2 hours with recombinant ESAT-6:CFP-10 or ESAT-6:CFP-10ΔC (7.5 µM), washed and incubated with 1 mg/ml (equivalent to 22.5 mM concentration) soluble OVA added exogenously for another 2 hours. Cells were washed, fixed and surface expression of SIINFEKL peptide (OVA_257–264_) on MHC-I was analysed as described earlier. PE conjugated isotype-matched Ab (BD Pharmingen, USA) was used as control.

### B3Z stimulation assay

Class I antigen presentation assay was carried out *in vitro* using B3Z (a kind gift from Dr. Satyajit Rath and Dr. Vineeta Bal, National Institute of Immunology, India), a CD8^+^ T cell hybridoma specific for OVA_257–264_ (SIINFEKL), presented on the murine H-2K^b^ MHC class I molecule [62] and using thioglycolate elicited peritoneal macrophages from C57BL/6 mice (H-2^b^) as antigen presenting cells (APCs). Macrophages either left untreated or pre-treated with 7.5 µM of ESAT-6:CFP-10 or ESAT-6ΔC:CFP-10 protein were loaded cytosolically with 10 mg/ml ovalbumin (with paraformaldehyde pre-fixed cells as well as BSA as control) in a hypertonic solution as described elsewhere [63]. Cells were washed and further incubated for 2 hours at 37°C. The OVA-loaded APCs (5×10^5^) were co-cultured with B3Z T cells (5×10^5^) in a 24-well tissue culture plate for 20–24 hours [64]. The IL-2 levels secreted in the culture supernatants were measured by ELISA.

### Ethics statement

All the animals studies were conducted following the guidelines laid by Committee for the Purpose of Control and Supervision of Experiments on Animals (CPCSEA), Ministry of environment and Forest, Govt. of India and performed in accordance with approved protocol (# P16/IAEC/NIN/2012/7/SG/) by the Institutional Animal Ethics Committee (IAEC) of National Institute of Nutrition, Hyderabad, India (Registration No. 154/1999/CPCSEA).

All the experiments involving human samples were approved by the Institutional Ethical Committee (IEC) of LEPRA Society-Blue Peter Research Centre, Hyderabad, India (IEC Project no. 10/2007). Written, informed consent was obtained from all subjects prior to sample collection.

### Statistical analyses

Data were expressed as mean ± SD of three independent experiments performed in a similar way. Groups were compared using the Kruskal-Wallis one-way ANOVA by ranks, followed by individual comparisons between the groups using the Mann-Whitney U Test. *p* value *of* ≤0.05 was considered to be significant. A statistics software package, IBM SPSS version 19.0 was used for all the statistical analyses.

## Supporting Information

Figure S1 **The DNA sequence of the clone 9.24 obtained from the yeast two hybrid screening and aligns with human β2M ORF.** The positive clone (9.24) sequence obtained from yeast two hybrid screening was queried against the Human genomic plus transcript database (Human G+T) using the NCBI MegaBlast algorithm. The Beta-2-microglobulin (β2M) was retrieved with the maximum score in the list of sequences producing significant alignment with 93% identity. The above alignment is the outcome from a pairwise local alignment of the clone 9.24 sequence and human β2M coding sequence (CDS) using the EMBOSS Water program with a gap open and gap extend penalty of 10 and 0.5 respectively.
(TIF)

Figure S2 **Structure of the ESAT-6 mutant (ESAT-6ΔC) with six amino acids deleted from the C-terminal end.** The C-terminal end of the ESAT-6 represents a poorly defined structure hanging away from the helical core. The deleted six amino acid residues (90–95) of ESAT-6ΔC are highlighted in red.
(TIF)

Figure S3 **ESAT-6:CFP-10 complex binds to β2M through the C-terminal end of ESAT-6.**The C-terminus of ESAT-6 is a structurally undefined region that is not involved in CFP-10 binding, deletion of 6 amino acids from the C-terminal end of ESAT-6 (ESAT-6ΔC) does not affect its binding to CFP-10, but the ESAT-6ΔC:CFP-10 complex fails to interact with β2M. The C-terminal end of ESAT-6 in the ESAT-6:CFP-10 complex is free and available for interaction with β2M.
(TIF)

Figure S4 **The ESAT-6:CFP-10 complex interacts with mouse β2M.** Recombinant His-tagged ESAT-6:CFP-10 protein was bound to Ni-NTA agarose beads and incubated for 2 hours with 1 mg cell lysate prepared from BMC2 mouse macrophages. After extensive wash the bound complexes were eluted by boiling in 1× Laemmli buffer. The samples were resolved on a 16% Tris-Tricine SDS-PAGE and transferred onto a nitrocellulose membrane and probed with rabbit anti-β2M Ab (Abcam, USA) followed by HRP conjugated anti-rabbit secondary Ab (Sigma-Aldrich, USA). Bands were visualized by addition of ECL reagent (GE Healthcare). Lane 1 is input control.
(TIF)

Figure S5 **ESAT-6 does not interact with β2M in complex with HLA class I.** PMA-differentiated THP-1 macrophage lysate was incubated with recombinant ESAT-6 or ESAT-6:CFP-10 protein. Mouse anti-human HLA-I Ab, clone HP1F7 (Santa Cruz Biotechnology) and Protein A/G agarose beads were used to pull down HLA-I α chain molecules from this mixture (Lanes 5 and 6). Control immunoprecipitation was carried out without the addition of anti-HLA-I Ab (Lanes 3 and 4). The protein A/G bound protein complexes were dissociated by boiling in 1× SDS-PAGE loading dye and immunoblotted for detecting ESAT-6 (Panel A) or β2M (Panel B) using either rabbit anti-His Ab or rabbit anti-human β2M Ab respectively. About 10% of the total lysate used in the pull down assays were used as input controls (Lanes 1 and 2). The blots were visualized by chemiluminescence after incubation with anti-rabbit IgG HRP conjugate. Results are representative of three different experiments.
(TIF)

Figure S6 **The recombinant ESAT-6:CFP-10 protein complex downregulates surface expression of β2M molecules.** PMA-differentiated THP-1 macrophages were treated with recombinant ESAT-6:CFP-10 complex protein for 2 hours at concentration of 7.5 and 12.5 µM. Cells were washed and incubated with either PE conjugated anti-human β2M or PE mouse IgM, κ isotype (BD Pharmingen) control antibody. β2M expression on cell surface was analyzed by flow cytometry. Results are representative of three independent experiments.
(TIF)

Figure S7 **The ESAT-6:CFP-10 complex is not cytotoxic to THP-1 macrophages.** PMA-differentiated THP-1 macrophages (2×10^5^/100 µl/well into a 96-well microplate) were treated with indicated concentrations of ESAT-6:CFP-10 for 2 hours. MTT (3-(4,5-dimethylthiazol-2-yl)-2,5-diphenyl tetrazolium bromide; Sigma-Aldrich) was added at a final concentration of 1 mg/ml for 4 hours after which cells were lysed with a lysis buffer (20% SDS in 50% dimethyl formamide) and the absorbance was recorded at 590 nm as described earlier (Khan *et al.*, 2008, Cell. Microbiol. 10:1711–1722).
(TIF)

Figure S8 **Treatment with ESAT-6:CFP-10 does not alter cell surface expression of other surface proteins.** PMA-differentiated THP-1 macrophages (A and B) or mouse thioglycolate elicited peritoneal exudate cells (C and D) were treated with 12.5 µM ESAT-6:CFP-10 complex for 2 hours after which surface expression of Mac-1 (A), TLR-4 (B), MHC-II (C) and CD14 (D) was measured by flow cytometry using specific primary and FITC conjugated secondary antibodies. Anti-Mac-1 Ab, anti-TLR4 Ab and anti-CD14 Ab were from BD Pharmingen and for staining MHC-II, supernatant from Y3P hybridoma cells was used. Results are representative of at least three different experiments.
(TIF)

Figure S9 **ESAT-6:CFP-10 or ESAT-6 alone does not change the expression of β2M at protein and mRNA levels.** (A) PMA-differentiated THP-1 macrophages were either left untreated or treated with two different concentrations of ESAT-6:CFP-10 for 2 hours and cell lysates were prepared. After protein estimation, 30 µg of lysates were separated on a 15% SDS-PAGE and transferred onto nitrocellulose membrane which was probed with anti-β2M Ab. β-actin levels were also measured in this blot to confirm equal protein loading. (B) HEK-293 cells were seeded at a density of 1×10^6^ in a 6 well plate and were transfected with either pEGFP-C1, pEGFP-C1-*esat-6* or pEGFP-C1-*esat-6ΔC* plasmid construct. After 20–24 hours, RNA was isolated from the transfected cells to synthesize cDNA. Specific primers were used for amplification of β2M and β-actin by PCR from the synthesized cDNA. Amplified products were resolved on a 1.5% agarose gel and visualized by ethidium bromide staining. Results are representative of three different experiments.
(TIF)

Figure S10 **Determination of purity of the enriched Rough Endoplasmic Reticulum (RER) fraction.** Equal amount of protein (15 µg per lane) extracted from the enriched RER fraction and whole cell lysate prepared from HEK-293 cells were separated on a 16% Tris-Tricine SDS-PAGE gel, transferred to a nitrocellulose membrane and the membrane was immunoblotted for the presence of β2M (ER-specific marker), EEA1 (endosome-specific marker), LAMP2 (lysosome-specific marker) and GAPDH (cytosol-specific marker) using appropriate combinations of primary and secondary Abs and visualized by chemiluminescence. Results are representative of at least three experiments.
(TIF)
