# Supplementary figures and images for: The ESAT-6 Protein of Mycobacterium tuberculosis Interacts with Beta-2-Microglobulin (β2M) Affecting Antigen Presentation Function of Macrophage
Source: PLoS Pathog. 2014 Oct 30;10(10):e1004446. doi: 10.1371/journal.ppat.1004446 (PMC4214792; doi:10.1371/journal.ppat.1004446)

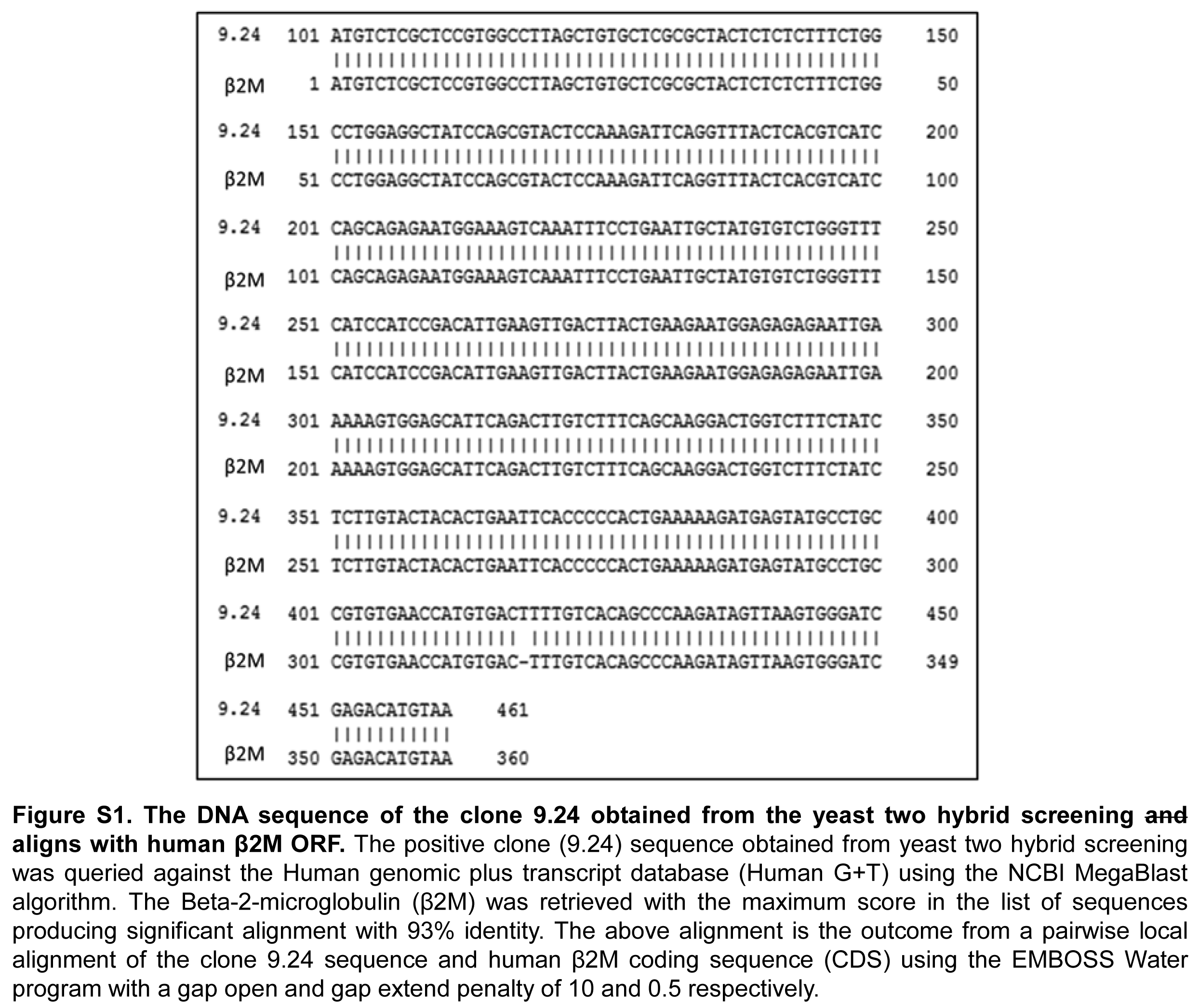

Supplement: Figure S1 — The DNA sequence of the clone 9.24 obtained from the yeast two hybrid screening and aligns with human β2M ORF. The positive clone (9.24) sequence obtained from yeast two hybrid screening was queried against the Human genomic plus transcript database (Human G+T) using the NCBI MegaBlast algorithm. The Beta-2-microglobulin (β2M) was retrieved with the maximum score in the list of sequences producing significant alignment with 93% identity. The above alignment is the outcome from a pairwise local alignment of the clone 9.24 sequence and human β2M coding sequence (CDS) using the EMBOSS Water program with a gap open and gap extend penalty of 10 and 0.5 respectively. (TIF) [file ppat.1004446.s001.tif]

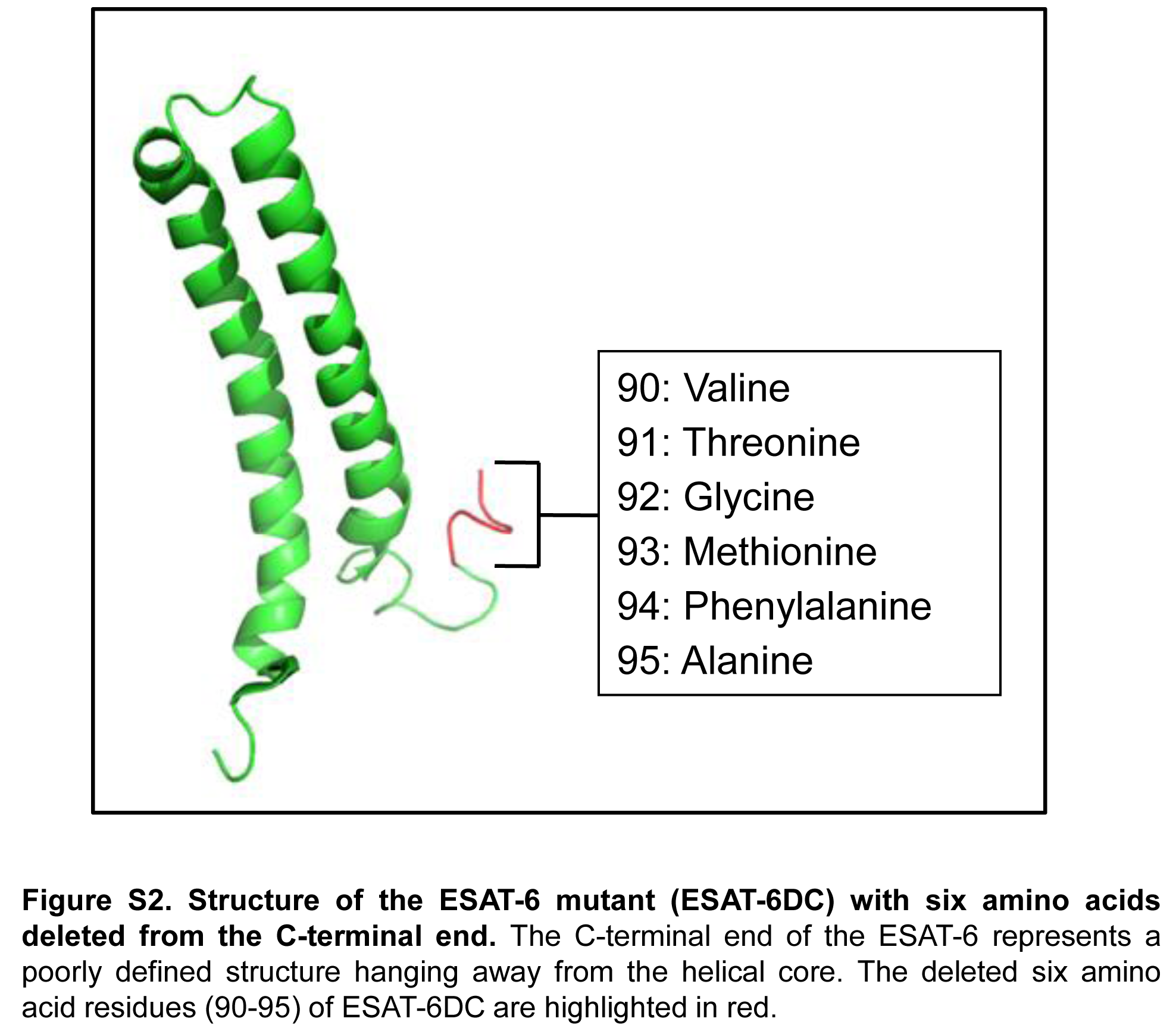

Supplement: Figure S2 — Structure of the ESAT-6 mutant (ESAT-6ΔC) with six amino acids deleted from the C-terminal end. The C-terminal end of the ESAT-6 represents a poorly defined structure hanging away from the helical core. The deleted six amino acid residues (90–95) of ESAT-6ΔC are highlighted in red. (TIF) [file ppat.1004446.s002.tif]

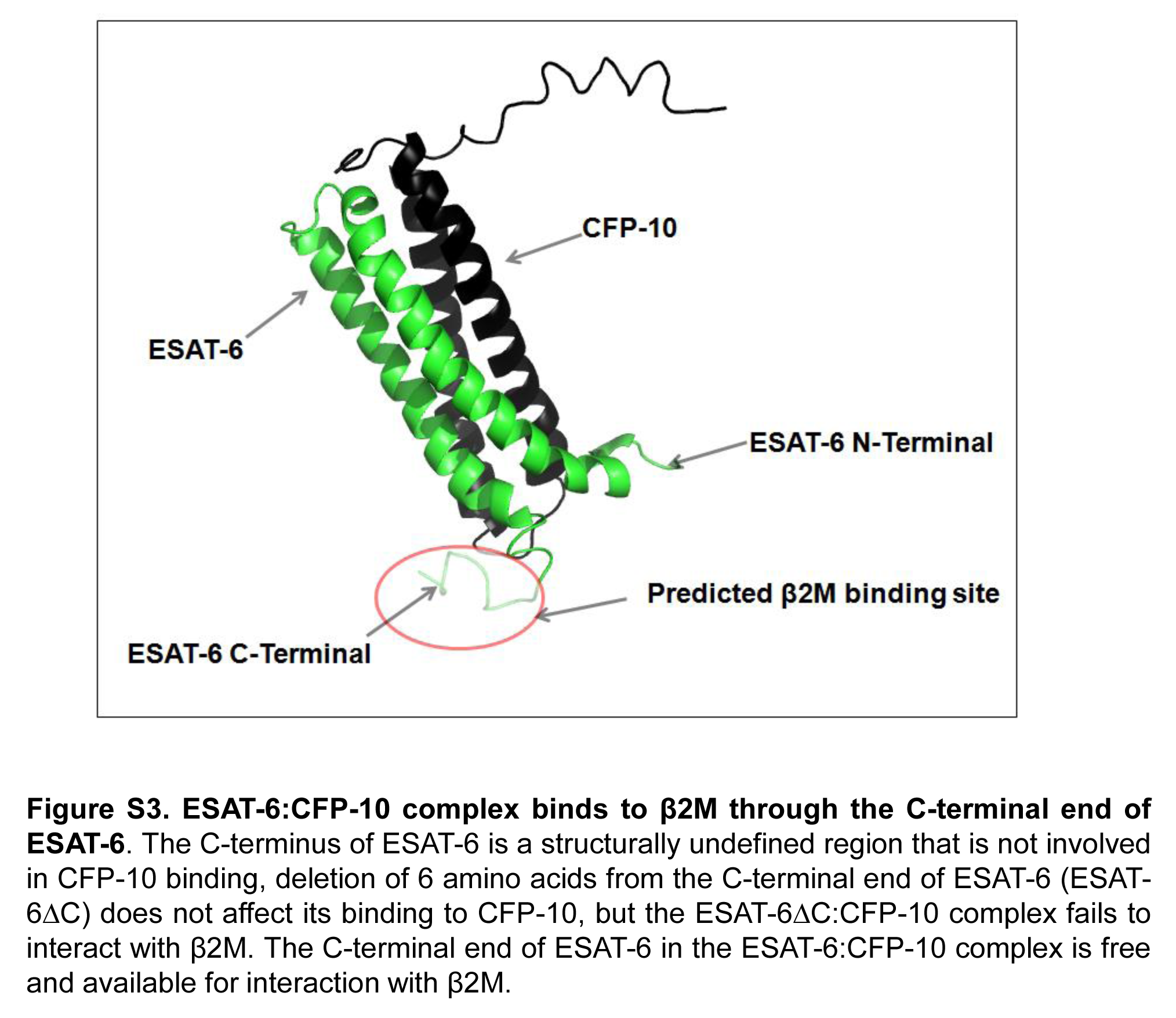

Supplement: Figure S3 — ESAT-6:CFP-10 complex binds to β2M through the C-terminal end of ESAT-6.The C-terminus of ESAT-6 is a structurally undefined region that is not involved in CFP-10 binding, deletion of 6 amino acids from the C-terminal end of ESAT-6 (ESAT-6ΔC) does not affect its binding to CFP-10, but the ESAT-6ΔC:CFP-10 complex fails to interact with β2M. The C-terminal end of ESAT-6 in the ESAT-6:CFP-10 complex is free and available for interaction with β2M. (TIF) [file ppat.1004446.s003.tif]

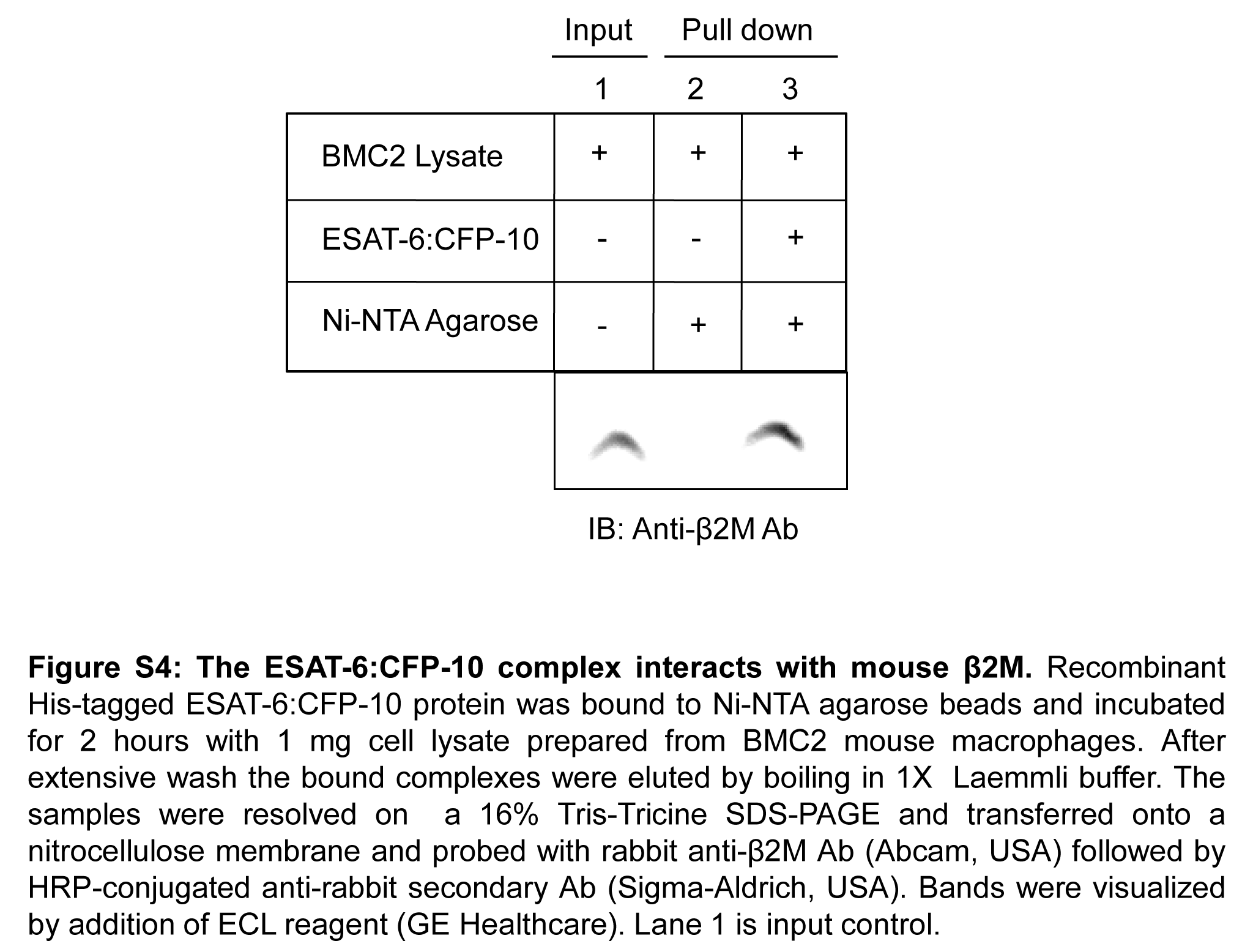

Supplement: Figure S4 — The ESAT-6:CFP-10 complex interacts with mouse β2M. Recombinant His-tagged ESAT-6:CFP-10 protein was bound to Ni-NTA agarose beads and incubated for 2 hours with 1 mg cell lysate prepared from BMC2 mouse macrophages. After extensive wash the bound complexes were eluted by boiling in 1× Laemmli buffer. The samples were resolved on a 16% Tris-Tricine SDS-PAGE and transferred onto a nitrocellulose membrane and probed with rabbit anti-β2M Ab (Abcam, USA) followed by HRP conjugated anti-rabbit secondary Ab (Sigma-Aldrich, USA). Bands were visualized by addition of ECL reagent (GE Healthcare). Lane 1 is input control. (TIF) [file ppat.1004446.s004.tif]

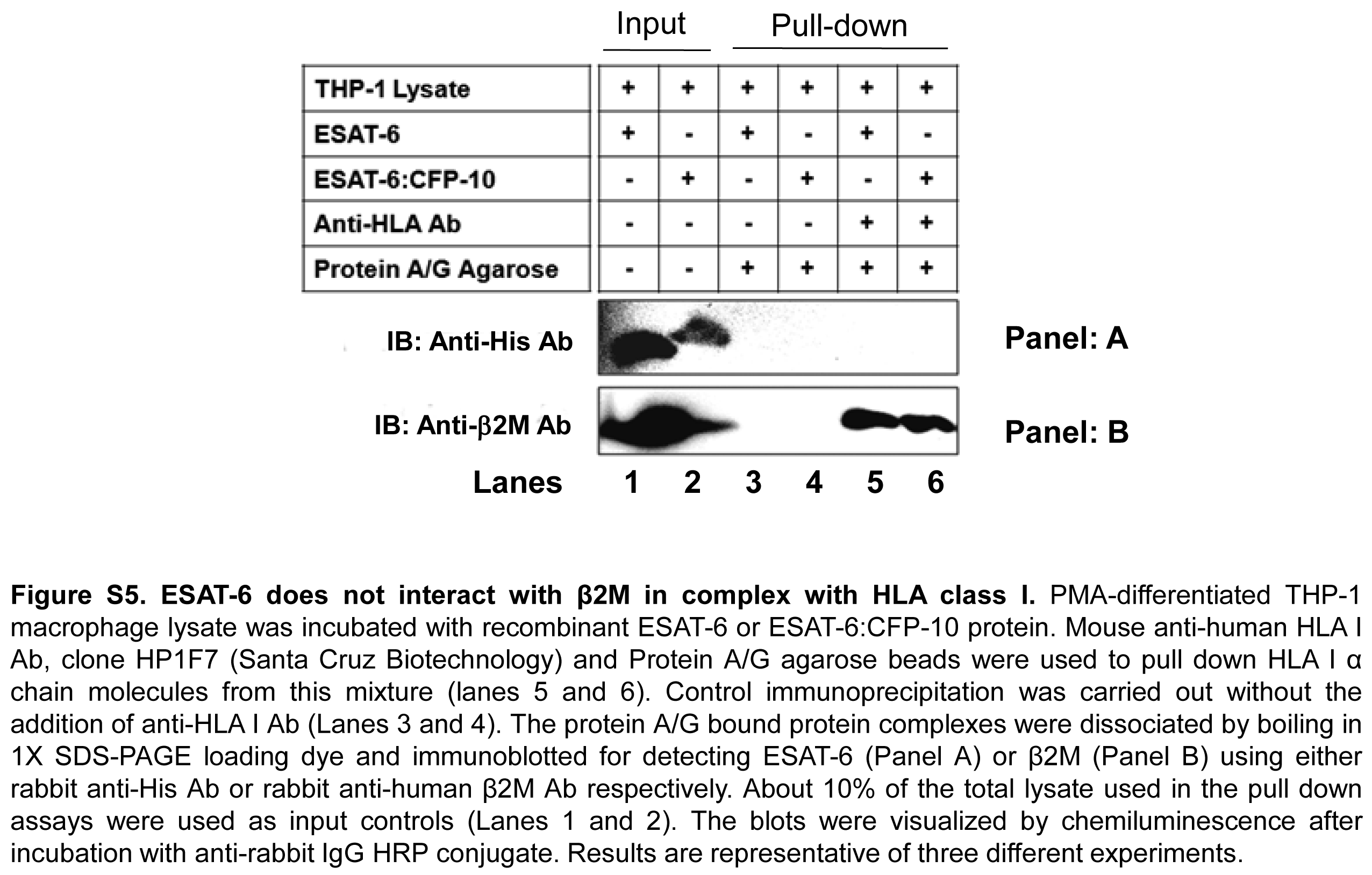

Supplement: Figure S5 — ESAT-6 does not interact with β2M in complex with HLA class I. PMA-differentiated THP-1 macrophage lysate was incubated with recombinant ESAT-6 or ESAT-6:CFP-10 protein. Mouse anti-human HLA-I Ab, clone HP1F7 (Santa Cruz Biotechnology) and Protein A/G agarose beads were used to pull down HLA-I α chain molecules from this mixture (Lanes 5 and 6). Control immunoprecipitation was carried out without the addition of anti-HLA-I Ab (Lanes 3 and 4). The protein A/G bound protein complexes were dissociated by boiling in 1× SDS-PAGE loading dye and immunoblotted for detecting ESAT-6 (Panel A) or β2M (Panel B) using either rabbit anti-His Ab or rabbit anti-human β2M Ab respectively. About 10% of the total lysate used in the pull down assays were used as input controls (Lanes 1 and 2). The blots were visualized by chemiluminescence after incubation with anti-rabbit IgG HRP conjugate. Results are representative of three different experiments. (TIF) [file ppat.1004446.s005.tif]

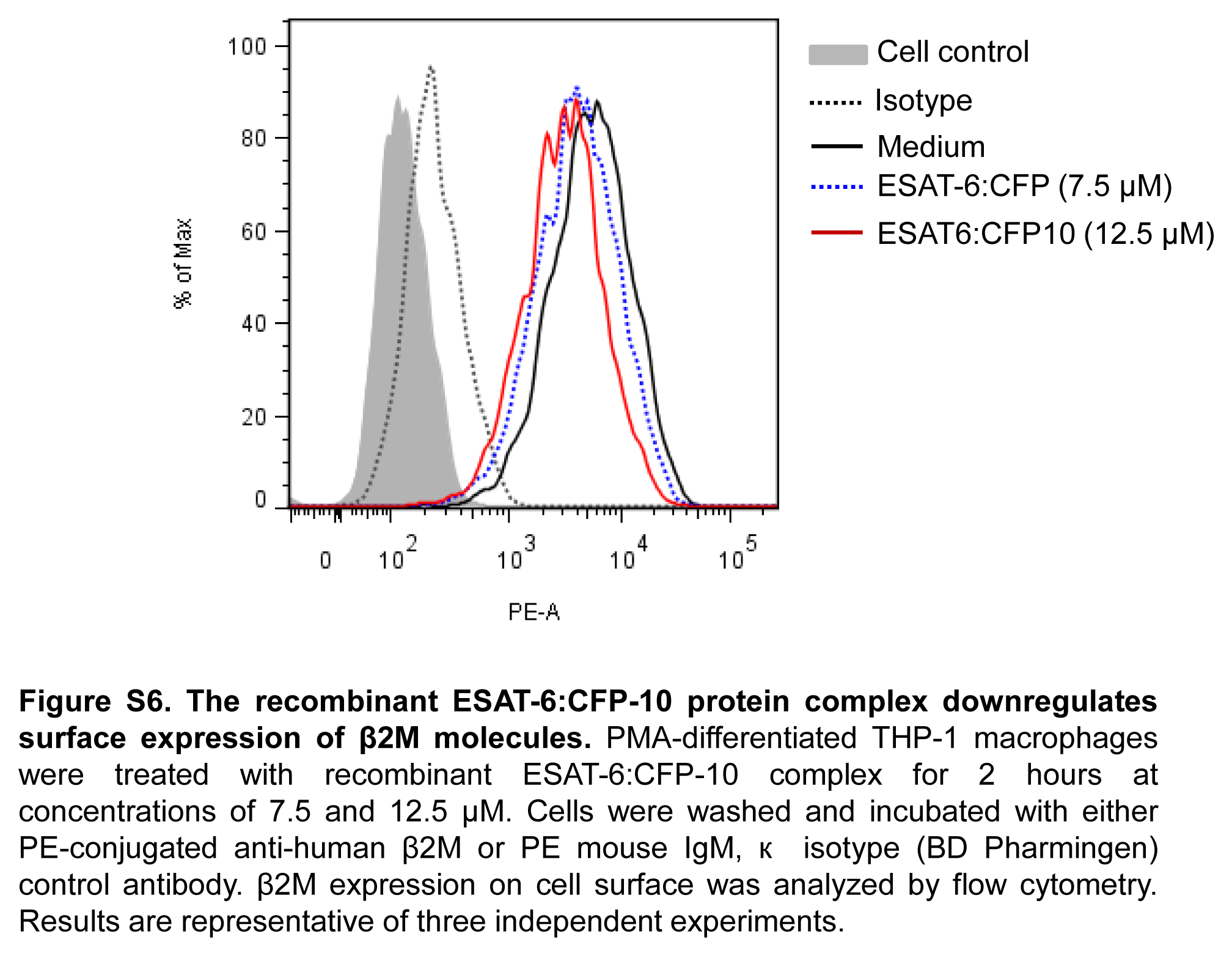

Supplement: Figure S6 — The recombinant ESAT-6:CFP-10 protein complex downregulates surface expression of β2M molecules. PMA-differentiated THP-1 macrophages were treated with recombinant ESAT-6:CFP-10 complex protein for 2 hours at concentration of 7.5 and 12.5 µM. Cells were washed and incubated with either PE conjugated anti-human β2M or PE mouse IgM, κ isotype (BD Pharmingen) control antibody. β2M expression on cell surface was analyzed by flow cytometry. Results are representative of three independent experiments. (TIF) [file ppat.1004446.s006.tif]

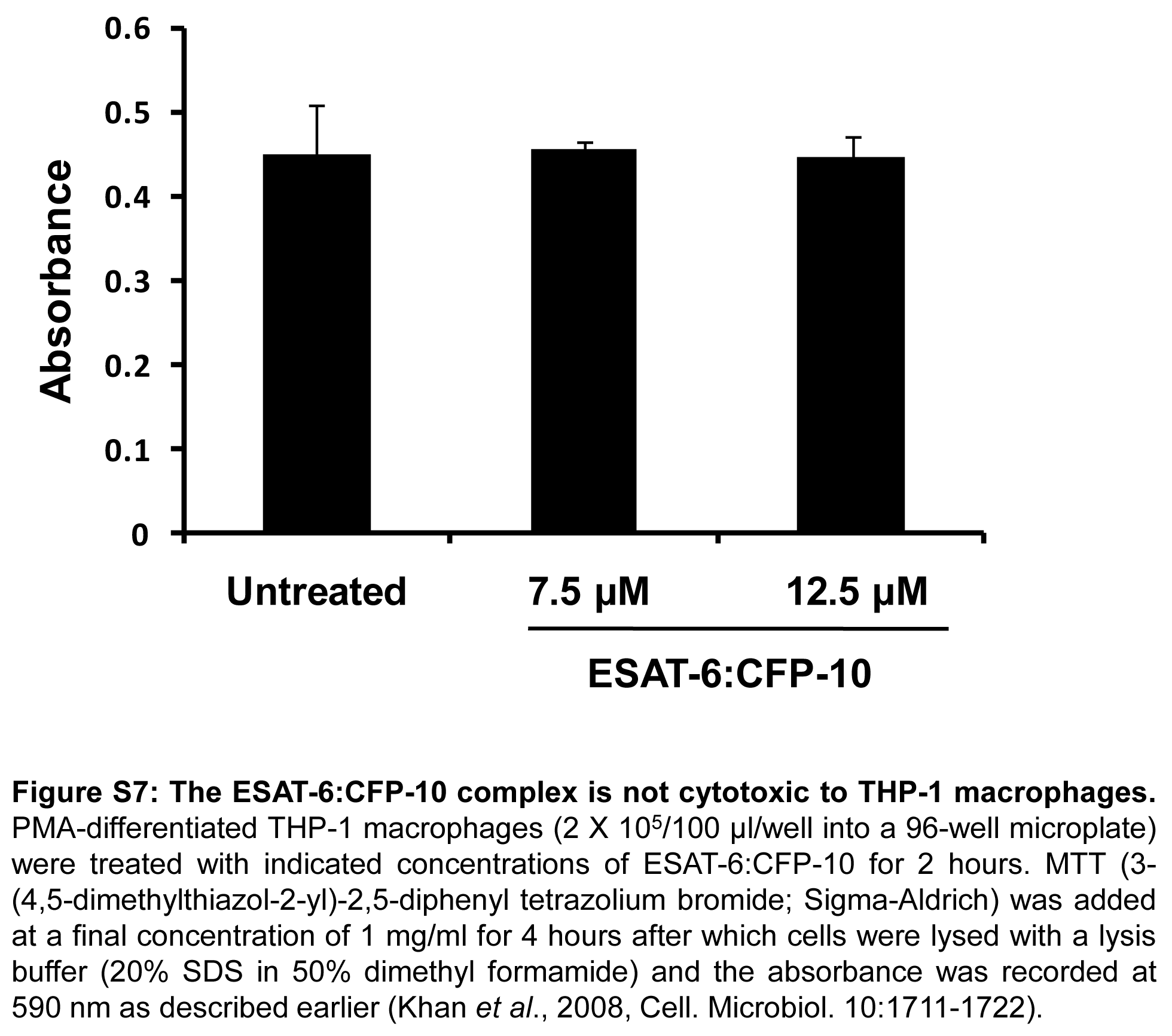

Supplement: Figure S7 — The ESAT-6:CFP-10 complex is not cytotoxic to THP-1 macrophages. PMA-differentiated THP-1 macrophages (2×105/100 µl/well into a 96-well microplate) were treated with indicated concentrations of ESAT-6:CFP-10 for 2 hours. MTT (3-(4,5-dimethylthiazol-2-yl)-2,5-diphenyl tetrazolium bromide; Sigma-Aldrich) was added at a final concentration of 1 mg/ml for 4 hours after which cells were lysed with a lysis buffer (20% SDS in 50% dimethyl formamide) and the absorbance was recorded at 590 nm as described earlier (Khan et al., 2008, Cell. Microbiol. 10:1711–1722). (TIF) [file ppat.1004446.s007.tif]

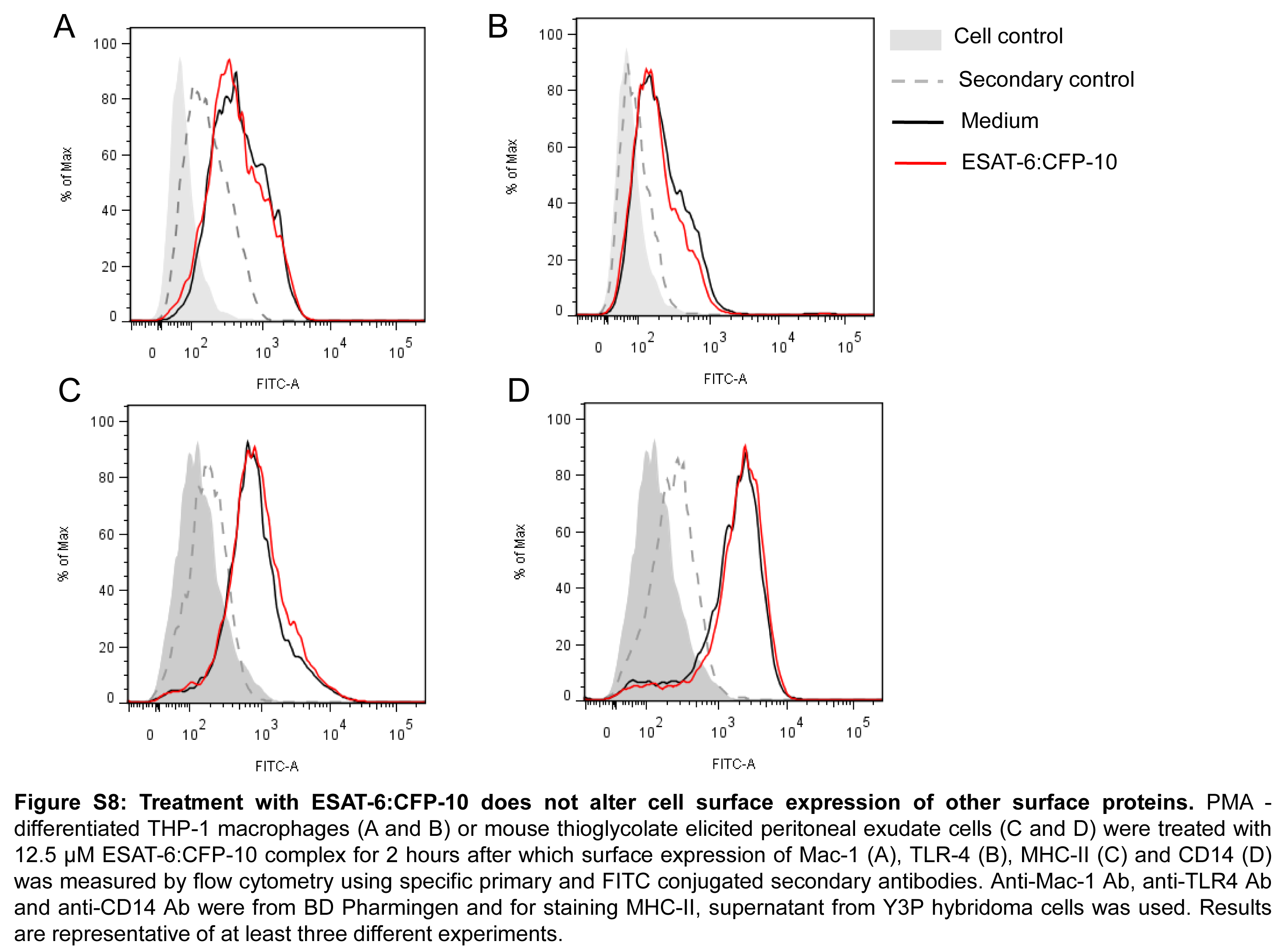

Supplement: Figure S8 — Treatment with ESAT-6:CFP-10 does not alter cell surface expression of other surface proteins. PMA-differentiated THP-1 macrophages (A and B) or mouse thioglycolate elicited peritoneal exudate cells (C and D) were treated with 12.5 µM ESAT-6:CFP-10 complex for 2 hours after which surface expression of Mac-1 (A), TLR-4 (B), MHC-II (C) and CD14 (D) was measured by flow cytometry using specific primary and FITC conjugated secondary antibodies. Anti-Mac-1 Ab, anti-TLR4 Ab and anti-CD14 Ab were from BD Pharmingen and for staining MHC-II, supernatant from Y3P hybridoma cells was used. Results are representative of at least three different experiments. (TIF) [file ppat.1004446.s008.tif]

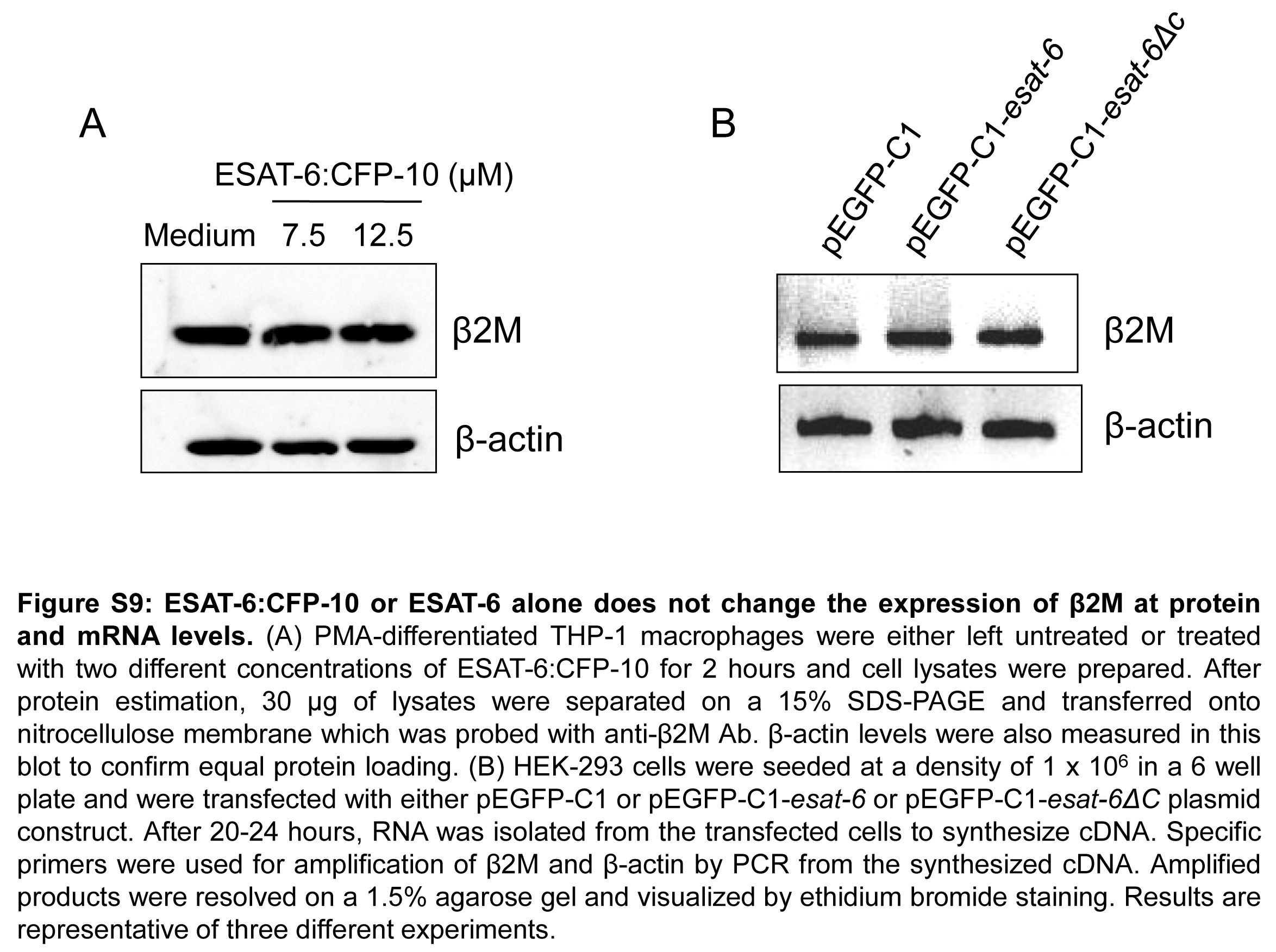

Supplement: Figure S9 — ESAT-6:CFP-10 or ESAT-6 alone does not change the expression of β2M at protein and mRNA levels. (A) PMA-differentiated THP-1 macrophages were either left untreated or treated with two different concentrations of ESAT-6:CFP-10 for 2 hours and cell lysates were prepared. After protein estimation, 30 µg of lysates were separated on a 15% SDS-PAGE and transferred onto nitrocellulose membrane which was probed with anti-β2M Ab. β-actin levels were also measured in this blot to confirm equal protein loading. (B) HEK-293 cells were seeded at a density of 1×10^6^ in a 6 well plate and were transfected with either pEGFP-C1, pEGFP-C1-esat-6 or pEGFP-C1-esat-6ΔC plasmid construct. After 20–24 hours, RNA was isolated from the transfected cells to synthesize cDNA. Specific primers were used for amplification of β2M and β-actin by PCR from the synthesized cDNA. Amplified products were resolved on a 1.5% agarose gel and visualized by ethidium bromide staining. Results are representative of three different experiments. (TIF) [file ppat.1004446.s009.tif]

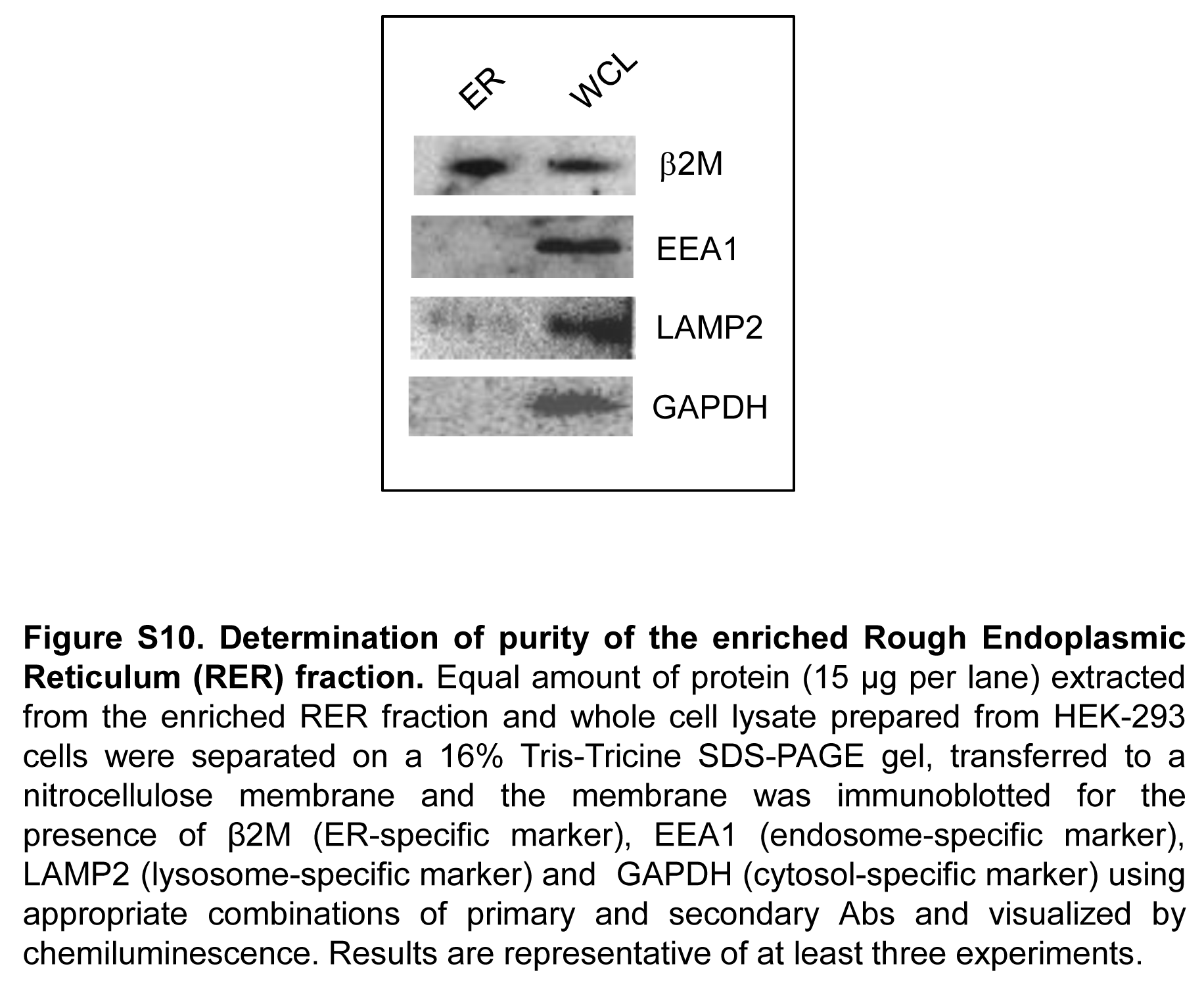

Supplement: Figure S10 — Determination of purity of the enriched Rough Endoplasmic Reticulum (RER) fraction. Equal amount of protein (15 µg per lane) extracted from the enriched RER fraction and whole cell lysate prepared from HEK-293 cells were separated on a 16% Tris-Tricine SDS-PAGE gel, transferred to a nitrocellulose membrane and the membrane was immunoblotted for the presence of β2M (ER-specific marker), EEA1 (endosome-specific marker), LAMP2 (lysosome-specific marker) and GAPDH (cytosol-specific marker) using appropriate combinations of primary and secondary Abs and visualized by chemiluminescence. Results are representative of at least three experiments. (TIF) [file ppat.1004446.s010.tif]
